# Insight into TPS-d Subfamily: The Evolutionary and Functional Diversity in Gymnosperms

**DOI:** 10.3390/biom16091318

**Published:** 2026-09-10

**Authors:** Sha Wang, Jinzhu Jiang, Sha Chen, Yan Liu, Cong Guo, Qingxia Xu, Jun Zhang, Siyuan Li, An Liu, Xianju Liu

**Affiliations:** 1 State Key Laboratory for Quality Ensurance and Sustainable Use of Dao-Di Herbs, Institute of Chinese Materia Medica, China Academy of Chinese Medical Sciences, Beijing 100700, China; wsha0718@163.com (S.W.); jzjiang@icmm.ac.cn (J.J.); schen@icmm.ac.cn (S.C.); yliu1980@icmm.ac.cn (Y.L.); cguo@icmm.ac.cn (C.G.); qxxu@icmm.ac.cn (Q.X.); jzhang@icmm.ac.cn (J.Z.); syli@icmm.ac.cn (S.L.); 2 Key Laboratory of Beijing for Identification and Safety Evaluation of Chinese Medicine, Institute of Chinese Materia Medica, China Academy of Chinese Medical Sciences, Beijing 100700, China

**Keywords:** terpenoids, TPS-d subfamily, evolution, function, gymnosperm

## Abstract

Gymnosperms represent an ancient lineage with diverse chemical profiles. Their terpenoids constitute a major class notable for substantial structural diversity. Terpene synthases (TPSs) are the driving force of terpene diversity. TPS-d is a gymnosperm-specific TPS subfamily, yet the evolution and functional diversity of TPS-d are still understudied. In this review, we analyze 134 functionally characterized gymnosperm TPSs to elucidate the evolutionary history and functional diversity of the predominant TPS-d subfamily. Phylogenetic analysis revealed substantial lineage-specific expansion and divergence within the TPS-d subfamily post-speciation. Crucially, we highlight the shifts in domain architecture during TPS-d evolution, providing structural evidence for the progressive attrition and eventual loss of the ancestral *γ* domain in TPS-d2 and TPS-d1 clades. Furthermore, we summarize the functional diversity of TPS-d enzymes in specialized metabolites, emphasizing their ecological roles in plant defense. Overall, this review provides a comprehensive overview of the evolutionary and functional landscape of the gymnosperm-specific TPS-d subfamily, serving as a valuable reference for future studies on TPSs in gymnosperms.

## 1. Introduction

Terpenoids are the largest and most structurally diverse class of natural products found in plants. To date, thousands of distinct terpenoid compounds have been identified [1]. In plants, a small subset of terpenoids function as primary metabolites indispensable for growth and development, including carotenoids for photosynthesis, as well as gibberellins, abscisic acid, and strigolactones as phytohormones [2,3,4,5,6]. The vast majority of terpenoids, however, are specialized metabolites that mediate ecological interactions and contribute to environmental adaptation and defense against herbivores and pathogens [7,8,9,10].

In addition to their ecological roles, terpenoids are major bioactive components in medicinal plants, with notable examples including the psychoactive and analgesic cannabinoids [11,12], the blockbuster anticancer drug paclitaxel [13], the frontline antimalarial agent artemisinin [14], and the cardiovascular-protective ginkgolide [15,16]. Due to the importance of terpenoids in biological and pharmaceutical significance, understanding the enzymes that generate terpenoid structural diversity has long been a central topic in plant biochemistry and metabolic engineering.

Terpenoids are derived from two common precursors: isopentenyl diphosphate (IPP) and dimethylallyl diphosphate (DMAPP), an allylic isomer of IPP. Their biosynthesis is conserved across the plant kingdom via two compartmentalized pathways: the cytosolic mevalonate (MVA) and the plastidial 2-C-methyl-D-erythritol-4-phosphate (MEP) pathways [17,18]. Subsequently, prenyltransferases (PTs) then catalyze the sequential condensation of IPP units to form geranyl diphosphate (GPP), farnesyl diphosphate (FPP), and geranylgeranyl diphosphate (GGPP) [19]. These molecules serve as the immediate substrates for terpene synthases (TPSs), the pivotal enzymes that orchestrate the transformation of linear precursors into a staggering array of hydrocarbon scaffolds through complex cyclization, dephosphorylation, and molecular rearrangement cascades.

Plant TPS proteins typically contain two conserved Pfam domains: PF03936 and PF01397 in Pfam models [20]. Based on their catalytic mechanisms and conserved motifs, TPS enzymes are classified into Class I, Class II, and bifunctional Class I/II [21]. Class I TPSs harbor conserved DDxxD and NSE/DTE motifs in the C-terminal *α*-domain and catalyze metal-dependent ionization of the diphosphate group. Class II TPSs contain a DxDD motif and initiate cyclization via protonation. Bifunctional Class I/II TPSs possess both active sites and perform a two-step cyclization cascade [22,23,24].

The rapid accumulation of genomic and transcriptomic data has led to the discovery of a growing number of *TPS* genes in gymnosperms [25,26,27,28]. However, systematic overview of the evolutionary history and functional diversification of the gymnosperm-specific TPS-d subfamily is still lacking. In this review, we synthesize current knowledge on 134 *TPS* genes from 27 gymnosperm species, reconstruct their phylogenetic relationships, reveal key evolutionary events, and summarize their functional roles in terpenoid biosynthesis and ecological roles. The aim of this review is to systematically clarify the evolutionary patterns and functional characteristics of the TPS-d subfamily in gymnosperms, thereby providing a clear research perspective for relevant follow-up studies.

## 2. Functional Diversity and Distribution of Characterized Gymnosperm TPSs

**Figure 1:**
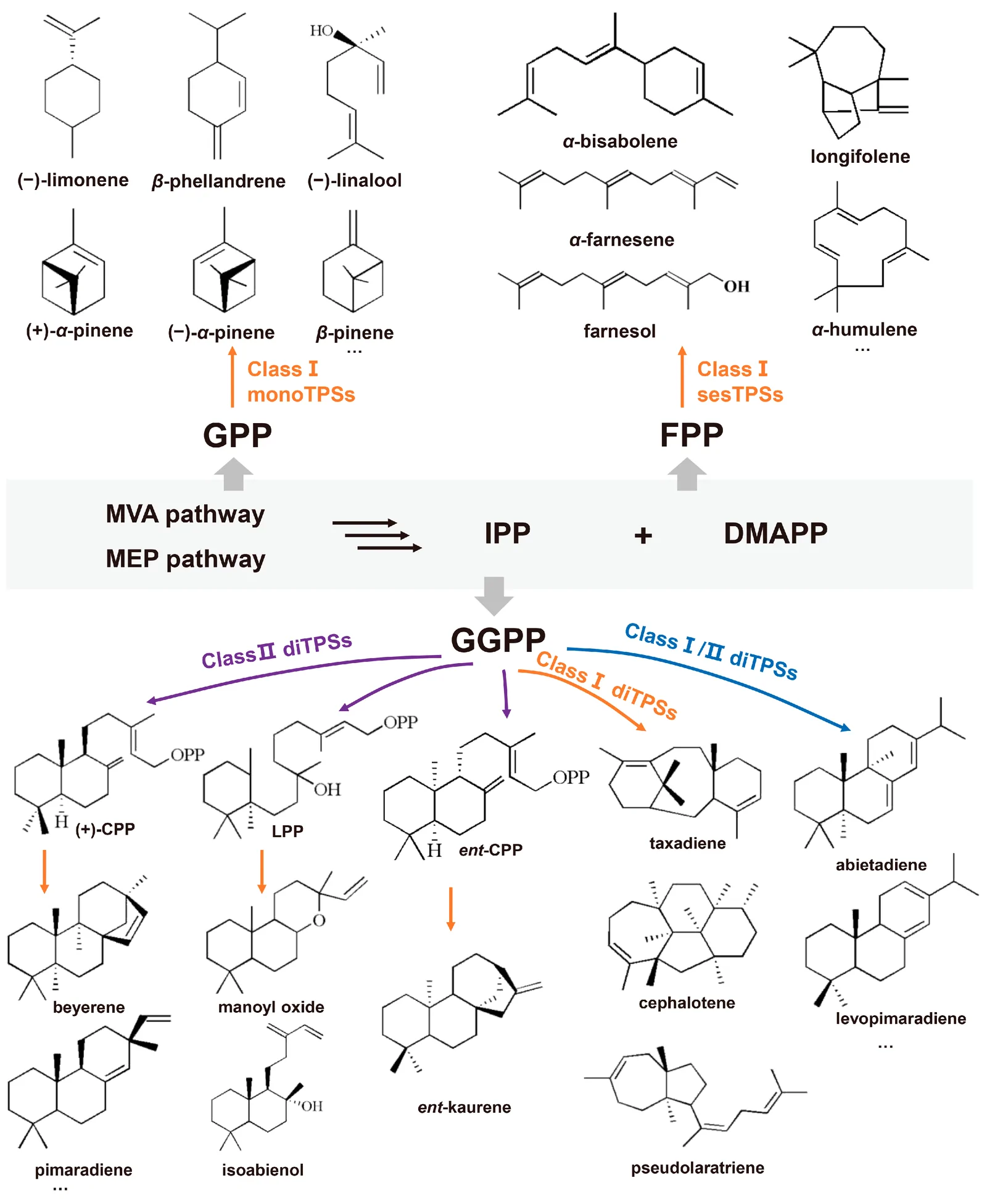
The diversity of TPSs in gymnosperms. Class I monoTPSs, sesTPSs and Class I, Class II, and Class I/II diTPSs have been extensively characterized in gymnosperms. The arrows in purple, orange, and blue represent Class II TPSs, Class I TPSs and Class I/II TPSs, respectively.

Terpenoids, as important active ingredients, are abundant in both gymnosperms and angiosperms [29,30,31,32]. As is known to all, TPSs are the first enzymes that generate the diverse structural framework of terpenoids [33,34]. In this study, we summarize 134 identified terpene synthases from 27 gymnosperm species, including 13 species of Cupressales, 13 of Pinales, and one species of Ginkgoales (Figure 1, Table S1). These TPSs are categorized into three major functional classes: monoterpene synthases (monoTPSs), sesquiterpene synthases (sesTPSs) and diterpene synthases (diTPSs). In summary, 54 TPSs utilized GPP as substrates to produce monoterpenes (Table S2), with *α*-pinene, *β*-pinene, *β*-phellandrene, (+)-3-carene and (−)-linalool being the most common products. In total, 19 TPSs catalyze FPP as substrates to produce sesquiterpenes (Table S3). A total of 61 diTPSs that use GGPP or intermediates such as (+)-CPP, *ent*-CPP, and LPP has been identified. These are further divided into Class I (33), Class II (14), and bifunctional Class I/II (14) diTPSs (Table S4). Of these, 14 Class II diTPSs produce key intermediates including (+)-CPP, *ent*-CPP, and LPP, whereas 25 Class I diTPSs then convert these intermediates into pimaradiene, beyerene, isopimaradiene and manoyl oxide. The remaining 8 Class I diTPSs directly catalyze GGPP to produce pseudolarene, cephalotene, and taxadiene, while 14 bifunctional Class I/II diTPSs directly convert GGPP into levopimaradiene, abietadiene, and *cis*-abienol (Figure 1).

## 3. Phylogenetic Analysis Reveals the Expansion and Distinction of TPS-d Subfamily

Terpenoids constitute the most chemically and structurally diverse class of natural products [23], a diversity primarily driven by the functional plasticity of terpene synthases (TPSs) [33]. The evolutionary expansion of the *TPS* gene family is largely attributed to gene duplication, which provides the genetic material necessary for neo- and sub-functionalization [35,36]. Additionally, domain loss and adaptive selection under environmental pressures have further enriched the functional landscape of this family [36]. Based on established nomenclature, plant *TPS* genes are categorized into seven subfamilies (TPS-a through TPS-h) [37]. Among these, TPS-h is unique to *Selaginella moellendorffii*, while TPS-a, -b, and -g are specific to angiosperms. In contrast, the TPS-d subfamily is strictly gymnosperm-specific [28].

To elucidate the evolutionary trajectory and functional specialization of gymnosperm TPSs, we performed a phylogenetic analysis of 134 validated protein sequences. The sequences were obtained from previous studies (Tables S2–S4), and the corresponding accession number were listed in Tables S2–S4. Then, the multisequence alignments of *TPS* genes were generated by employing the ClustalW program with default parameters. The maximum-likelihood (ML) tree was constructed using MEGA (v11.0) with 1000 bootstrap replicates and was visualized by iTOL (https://itol.embl.de/ (accessed on 1 July 2026)).

**Figure 2:**
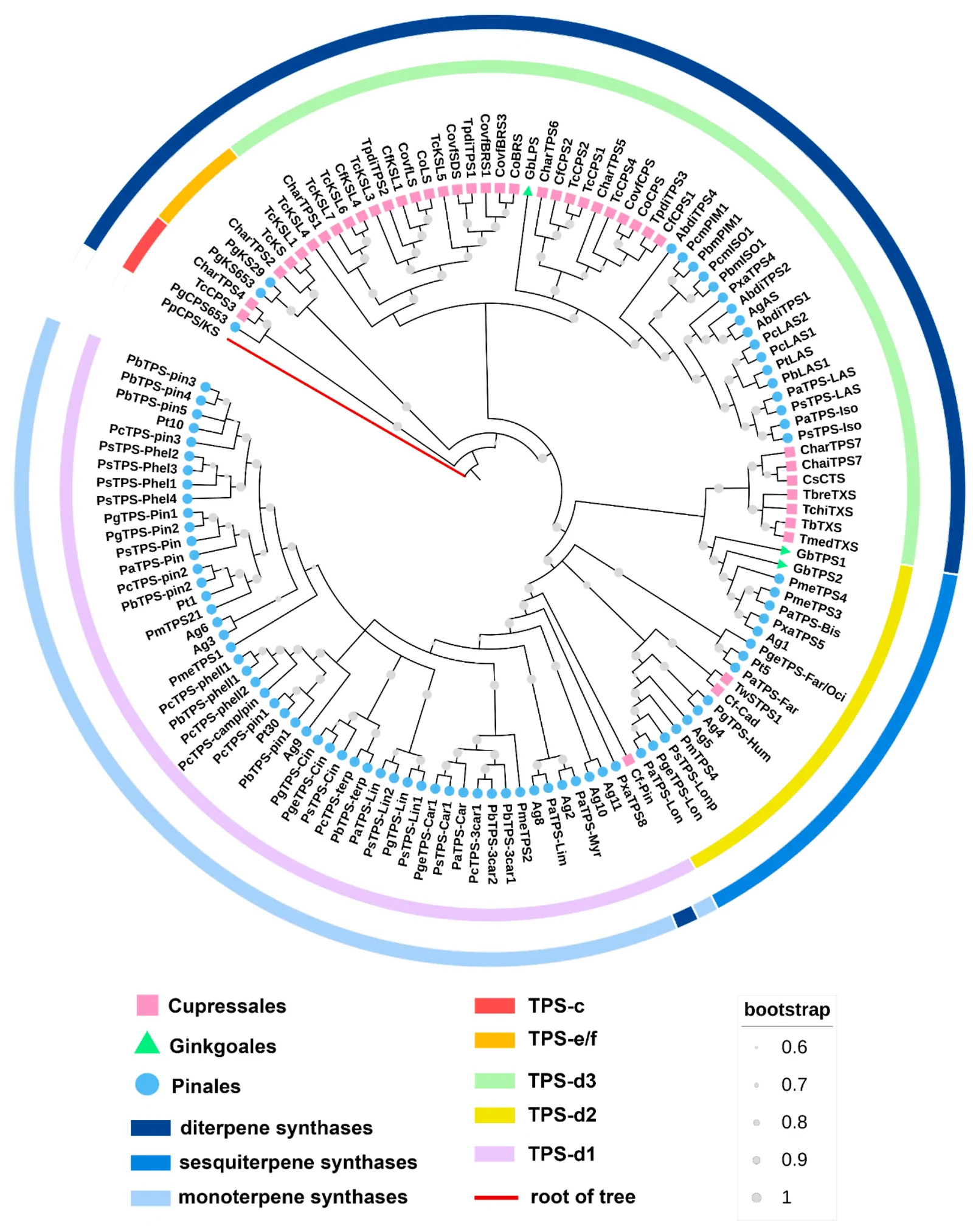
Phylogenetic tree of the gymnosperm-specific TPS-d subfamily. The tree is rooted with the bifunctional diTPS *ent*-CPS/*ent*-kaurene from *Physcomitrella patens* (*PpCPS*/*KS*). Each dot in gray represents bootstrap support of >60% (1000 repetitions). The inside ring features color-coded subfamilies and clades: red represents TPS-c subfamily, orange represents TPS-e/f subfamily, light green represents TPS-d3 clade, yellow represents TPS-d2 clade, and light purple represents TPS-d1 clade. A circle filled with blue represents Pinales members, a triangle filled with green represents Ginkgoales members, and a square filled with pink represents Cupressales members. For the outside ring, dark blue represents diterpene synthases, blue represents sesquiterpene synthases, and light blue represents monoterpene synthases. The branch in red indicates the root of this tree. Abbreviation: Pp, *Physcomitrium patens*; Cs, *Cephalotaxus sinensis*; Chai, *Cephalotaxus hainanensis*; Char, *Cephalotaxus harringtonia*; Ab, *Abies balsamea*; Ag, *Abies grandis*; Pxa, *Pseudolarix amabilis*; Pa, *Picea abies*; Pb, *Pinus banksiana*; Pc, *Pinus contorta*; Cf, *Chamaecyparis formosensis*; Tp, *Thuja plicata*; Tc, *Taiwania cryptomerioides*; Gb, *Ginkgo biloba*; Co, *Chamaecyparis obtusa*; Cov, *Chamaecyparis obtusa* var. *formosana*; Pg, *Picea glauca*; Ps, *Picea sitchensis*; Pme, *Pseudotsuga menziesii*; Tmed, *Taxus media*; Tb, *Taxus baccata*; Tbre, *Taxus brevifolia*; Tchi, *Taxus chinensis*; Pge, *Picea engelmannii* × *Picea glauca*; Pt, *Pinus taeda*; Pm, *Pinus massoniana*; Tw, *Taxus wallichiana*.

The maximum-likelihood tree, anchored by the ancestral bifunctional diTPS *PpCPS/KS* from *Physcomitrium patens* [38], resolved three major clades: TPS-c, TPS-e/f, and TPS-d (Figure 2). Within the gymnosperm-specific lineage, the TPS-d subfamily further partitioned into three well-supported subclades: TPS-d1, TPS-d2, and TPS-d3 (Figure 2). Phylogenetic analysis is also built by aligned encoded amino acid sequences of identified gymnosperms TPS genes alongside those from *Arabidopsis thaliana* and *Solanum lycopersicum* (Figure S1, the accession numbers of these TPSs are shown in Table S5), highlighting the TPS-d clade is exclusive to gymnosperms.

### 3.1. Conserved TPS-c and TPS-e/f Genes Dominate Primary Metabolism with Functional Divergence

Our analysis highlights a contrast in evolutionary dynamics between these subfamilies. The TPS-c and TPS-e/f subfamilies appear highly conserved in gymnosperms with limited expansion, currently comprising only three and five identified members, respectively. Conventionally, these subfamilies harbor enzymes dedicated to primary metabolism, specifically *ent*-CPP synthase and *ent*-kaurene synthase in the gibberellic acid pathway [39,40,41].

However, functional “leakage” into specialized metabolism is observed, as exemplified by TcKSL1 from *Taiwania cryptomerioides*, which belongs to the TPS-e/f clade yet produces the specialized metabolite biformene [40]. Notably, biformene can also be synthesized by CharTPS1, a member of the TPS-d subfamily [39], illustrating how similar chemical phenotypes in gymnosperms can arise through convergent or species-specific evolution across different subfamilies. Indeed, certain members of the TPS-c and TPS-e/f subfamilies can also catalyze the synthesis of specialized metabolites [42]. For example, the TPS-f genes in angiosperms, SlyTPS46 from *Solanum lycopersicum*, used GGPP to produce geranyl linalool [43]. The TPS-c member CusTPS23, in *Cucumis sativus*, produces myrcene and linalool from GPP, and catalyzes FPP to produce *β*-farnesene and nerolidol [44]. In gymnosperms, the sole exception TcKSL1 demonstrates that the TPS-c and TPS-e/f subfamilies are more conserved and predominantly involved in primary metabolism.

### 3.2. TPS-d Subfamily Involved in Secondary Metabolism Experiences Rapid Expansion

In contrast, the TPS-d subfamily has undergone massive expansion, accounting for 125 of the 134 characterized gymnosperm TPSs. This expansion is functionally structured across the three subclades with notable specialized exceptions. Within the TPS-d1 clade, which comprises 54 members, the majority of enzymes function as monoterpene synthases; however, this group also includes PxaTPS8, a specialized diterpene synthase responsible for pseudolarene biosynthesis [45]. This functional shift illustrates the dynamic nature of substrate recruitment in gymnosperm TPS evolution, confirming that phylogenetic proximity does not always dictate enzymatic product specificity.

Similarly, the TPS-d2 clade, containing 19 members, is characterized by sesquiterpene synthases such as longifolene and bisabolene synthases. The TPS-d3 clade, representing the remaining 52 members, consists entirely of specialized diterpene synthases.

Notably, TPS sequences from the Cupressaceae and Pinaceae form distinct, family-specific clusters, suggesting that lineage-specific expansion might occurred independently within these families after their evolutionary divergence (Figure 2). Previous comparative genomic studies have shown that lineage-specific gene duplication, particularly tandem duplication, has contributed substantially to the expansion of the gymnosperm-specific TPS-d subfamily [28]. Our tree suggested this lineage-specific expansion, which is observed across a broader range of gymnosperms, might been predominantly driven by lineage-specific tandem duplications.

This pattern of family-specific diversification, underpinned by these lineage-specific duplication events and subsequent functional divergence, highlights the modular nature of TPS evolution in gymnosperms [28]. Ultimately, these evolutionary dynamics might have driven the adaptive tailoring of specialized terpenoid profiles, allowing different gymnosperm lineages to effectively meet their unique ecological and physiological demands [36,46,47].

## 4. Domain Losing Occurred in Gymnosperm Terpene Synthase Evolution

**Figure 3:**
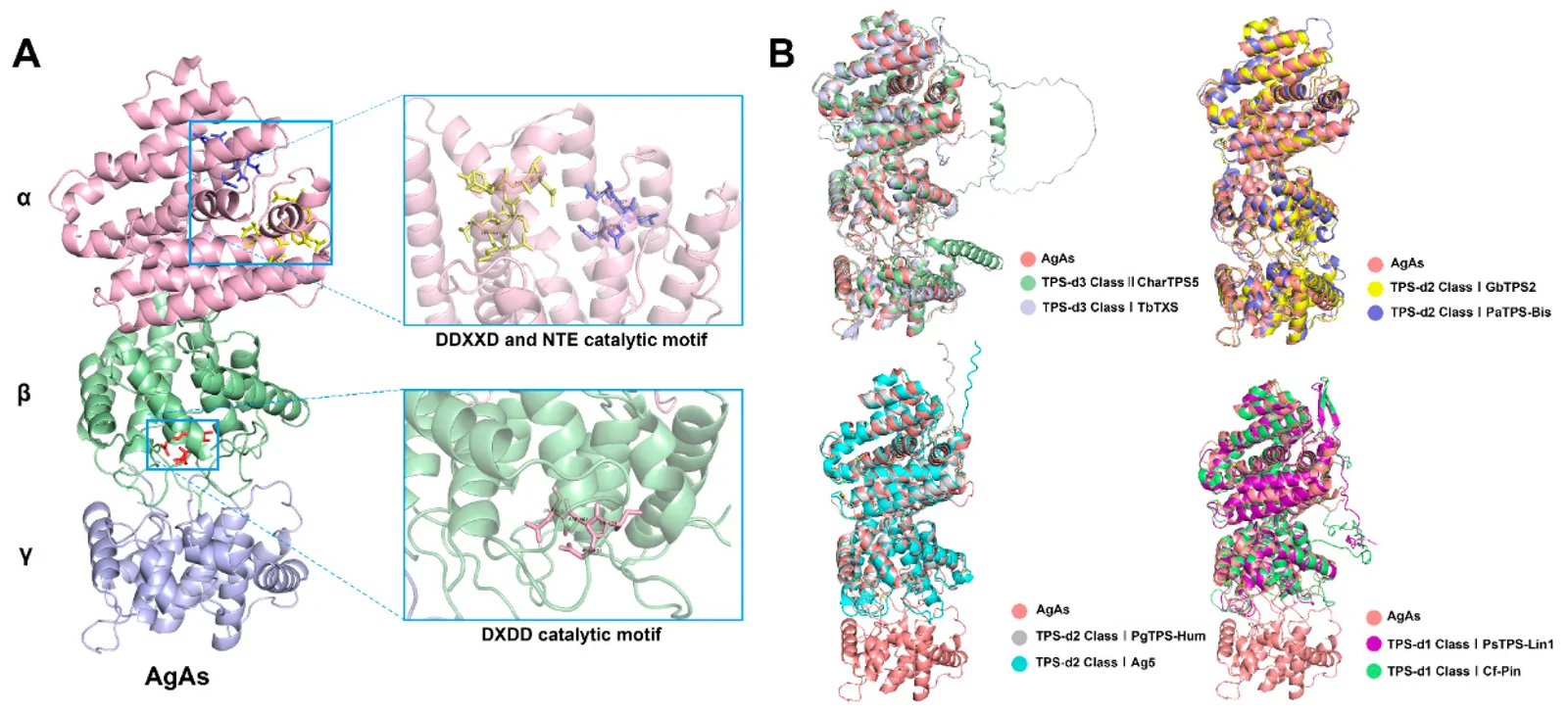
The domain architecture of TPS-d3, TPS-d2 and TPS-d1 members reveals the progressive loss of the *γ* domain within the TPS-d subfamily. (**A**) Crystal structure of AgAS. The *α* domain (residues 559–868), *β* domain (residues 110–133, 350–558), and *γ* domain (residues 134–349) domains are shown in light pink, light green, and light blue, respectively. The Class II DXDD catalytic motif corresponds to residues 402 to 405, while the Class I catalytic motifs DDXXD and NTE correspond to residues 621–625 and residues 765–773, respectively. (**B**) The alignment of AgAs with TPS-d3 Class I and Class II TPSs revealed that both enzyme types retain the *αβγ* tridomain. When aligned with TPS-d2 Class I TPSs, AgAs shows that only some members of this group maintain the *αβγ* tridomain, whereas others lack the *γ* domain. In contrast, alignment with TPS-d1 Class I TPSs demonstrated that all TPS-d3 enzymes possess only the *αβ* bidomain, having lost the *γ* domain entirely.

The structural streamlining of TPS enzymes, specifically through the loss of the ancestral *γ*-domain, has been a decisive factor in the functional diversification of the plant TPS family [48]. To investigate domain evolution in gymnosperms, we predicted the 3D structures of 129 TPS proteins by AlphaFold3 [49], and compared them with the archetypal *γβα*-tridomain structure of *Abies grandis* abietadiene synthase (AgAS). The predicted protein structures are showed in Figures S2–S4. Structural comparisons revealed that all TPS-d3 members retain the complete *γβα* tridomain architecture (Figure 3). Interestingly, the diterpene synthase PxaTPS8 within the TPS-d1 clade retains the full tridomain architecture, distinguishing it from its monoterpene-producing relatives. Together, these results support that all diTPSs in gymnosperms retain a tridomain structure (Figure S2).

Furthermore, structural analysis uncovered that multiple TPS-d2 proteins, including Ag1, GbTPS1, GbTPS2, PaTPS-Bis, PmeTPS3, PmeTPS4 and PxaTPS5, sharing the identical *γβα* tridomain architecture (Figure S3). Nevertheless, all of these enzymes lack the conserved DxDD motif, a deletion that abolishes their Class II catalytic activity. Taken together, these results demonstrate that the DxDD motif is an essential structural determinant conferring Class II catalytic function to gymnosperm TPSs.

In addition, structural analysis of monoTPSs within the TPS-d1 clade shows the loss of the *γ* domain. These changes in domain architecture show that a *γ* domain was progressively lost in both the TPS-d2 and TPS-d1 clades, and this loss ultimately led to the emergence of the TPS-d1 clade. Furthermore, this discrepancy between physical structure and biochemical function provides explicit evidence that the loss of key catalytic motifs preceded the actual physical deletion of the *γ*-domain.

**Figure 4:**
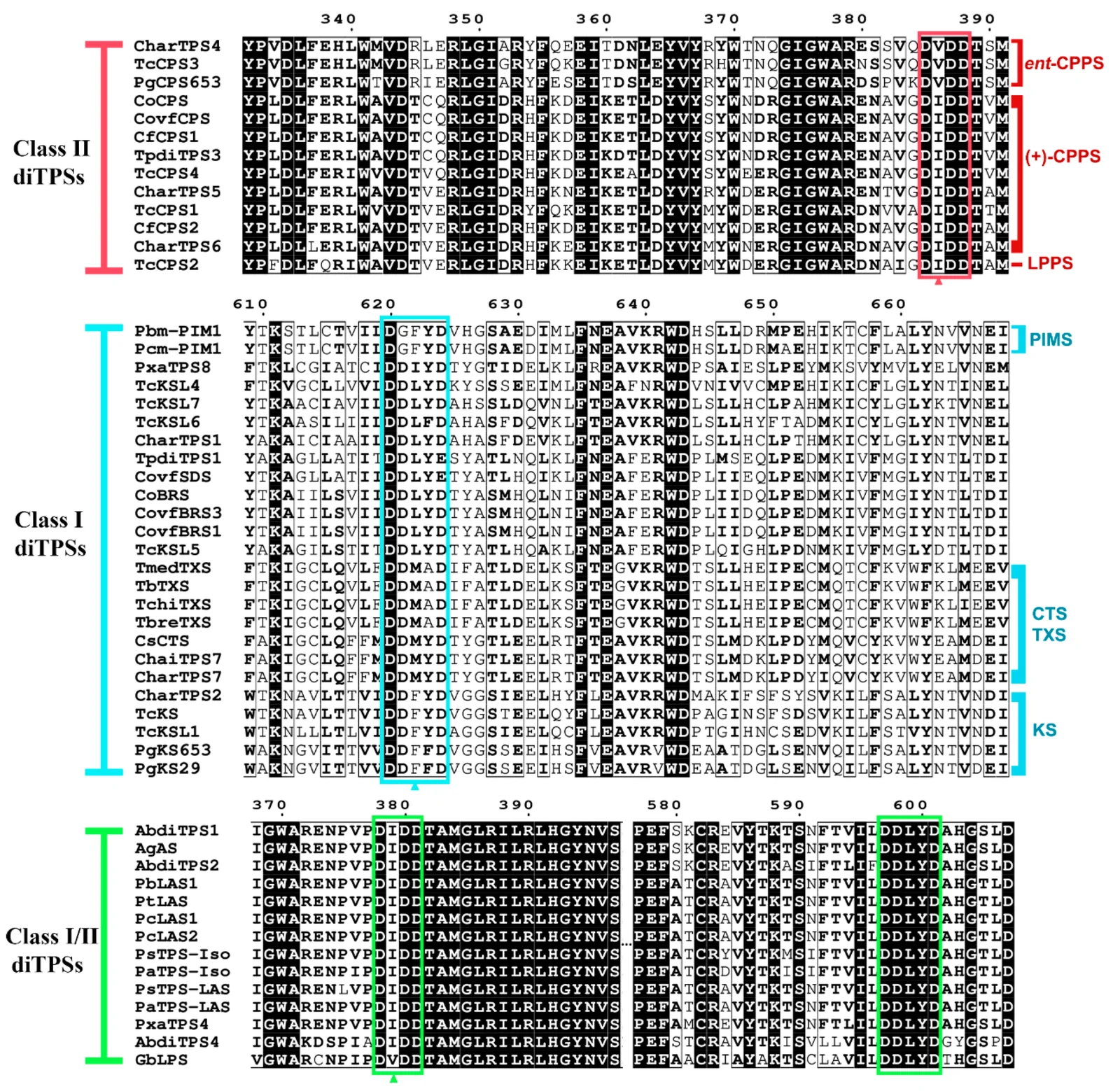
The motif features of Class I, Class II and Class I/II terpene synthases in gymnosperms. Multiple sequence alignment of gymnosperm diTPSs is shown. The signature motifs of Class I (DDxxD) and Class II (DxDD) enzymes are shown in solid boxes. The triangles under the boxes represent different conserved sites in diTPS synthases. In Class II type TPSs, *ent*-CPS contains a DVDD motif, while (+)-CPS and LPS contain a DIDD motif. In Class I type TPSs, the TS-like synthase has a DDMAD site, the CTS have a DDMYD site, the *ent*-KS synthase has a DDF(F/Y)D site, and the pimaradiene synthase has a DGFYD site. The abbreviations of the synthases are the same as Table S1.

Conserved motif analysis further supported functional classification. Gymnosperm TPSs exhibit highly conserved active sites tailored to their respective catalytic classes. Bifunctional Class I/II TPSs, including AgAS, AbdiTPS1/2/4, and PbLAS1, possess the characteristic DIDD and DDLYD motifs required for tandem cyclization (Figure 4). Within Class II TPSs, the active site for (+)-copalyl diphosphate (CPS) synthesis is defined by a DIDD motif, whereas the production of *ent*-CPS is distinguished by a DVDD variant (Figure 4). In Class I TPSs, the DDLYD motif is predominant, though further specialization is observed: taxadiene-like synthases (TS-like) and cephalotene synthases (CTS) typically harbor DDM(A/Y)D sites, while *ent*-kaurene synthases (*ent*-KS) feature a DDF(F/Y)D signature. Moreover, pimaradiene synthases in *Pinus contorta* and *P. banksiana* are characterized by a unique DGFYD motif (Figure 4). Although most TPS-d1 and d2 members have transitioned to Class I enzymes via the loss of the DxDD motif, they retain other class-specific signatures; for instance, an RR(X)8W motif is highly conserved near the N-terminus of most monoTPSs and sesTPSs (Figures S5 and S6), but is conspicuously absent in diTPSs (Figure S7).

## 5. Gymnosperm-Specific TPS-d Subfamily Is Responsible for Specialized Terpenoids Biosynthetic

Throughout their evolutionary history, *TPS* genes have undergone extensive lineage-specific duplication and divergence, culminating in the functional diversification observed across different subfamilies [42]. In gymnosperms, the TPS family is categorized into three major clades: TPS-d, TPS-c, and TPS-e/f. While the TPS-c and TPS-e/f subfamilies have been extensively characterized in angiosperms as key players in specialized metabolism [42], their counterparts in gymnosperms appear predominantly involved in primary metabolism, as evidenced by the functional characterization of eight TPS-c and TPS-e/f members [39,40,41]. A notable exception is TcKSL1 from Taxus chinensis, which belongs to the TPS-e/f clade yet is specialized for the production of the secondary metabolite biformene [40].

The gymnosperm-specific TPS-d subfamily is exclusively responsible for specialized terpenoids. As shown by the phylogenetic analysis described above, the TPS-d subfamily has undergone significant expansion and divergence (Figure 2).

### 5.1. TPS-d1 Clade Predominantly Governs Monoterpene Synthesis with a Distinct diTPS Exception

**Figure 5:**
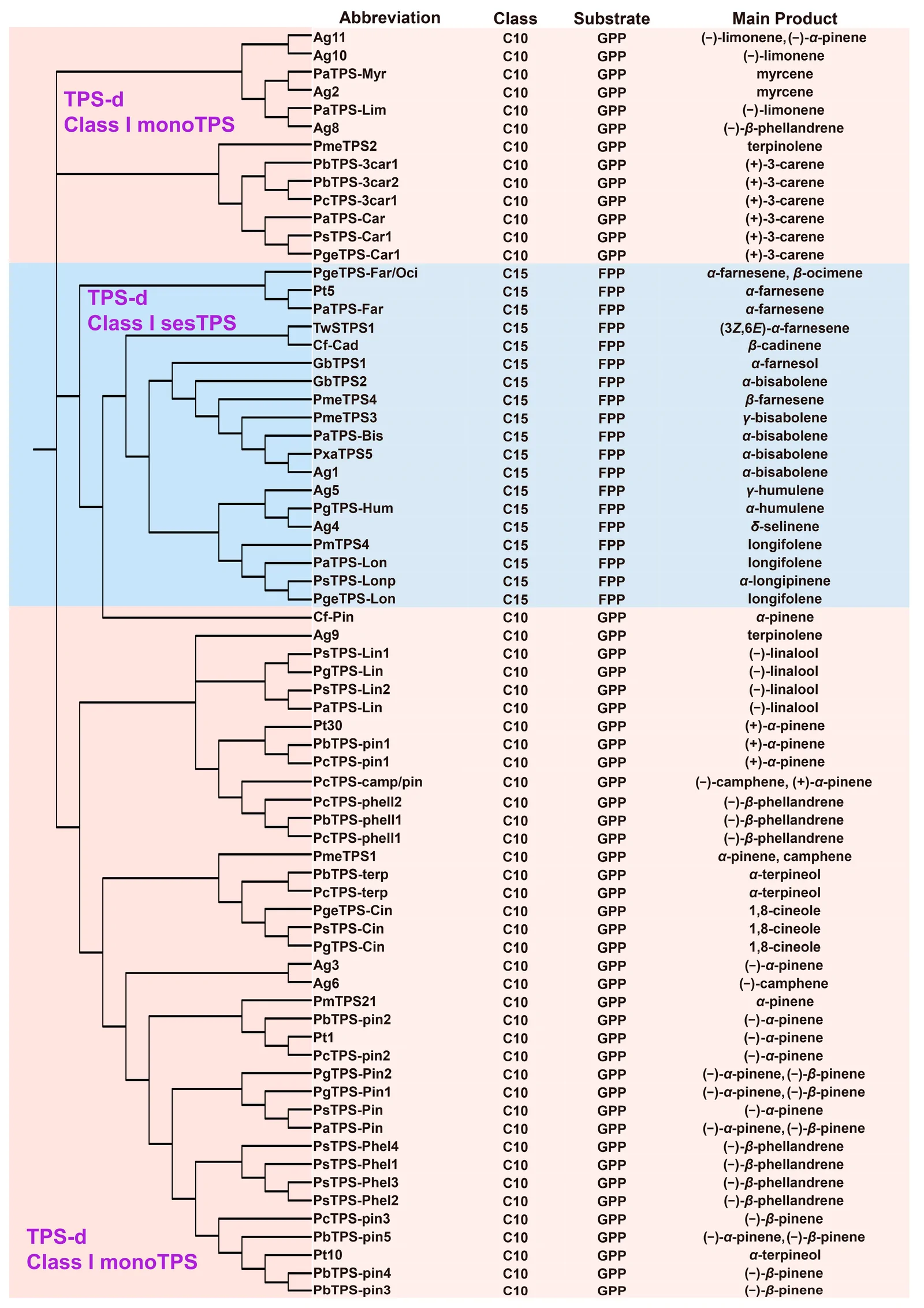
Phylogenetic analysis and functional characteristics of monoTPSs and sesTPSs in TPS-d genes. The left panel is a maximum-likelihood phylogenetic tree of sesTPSs and monoTPSs genes. In the right panel, Class I monoTPS and sesTPS genes are distinguished by different colors. The Class I monoTPS and sesTPS genes are shaded in pink and blue, respectively. The four columns show the abbreviation, product class, substrate, and major product, respectively. FPP, farnesyl diphosphate; GPP, geranyl diphosphate.

TPS-d1 clade predominantly comprises Class I monoterpene synthases (monoTPSs), which are the primary architects of gymnosperm essential oils and oleoresin volatiles (Figure 5). A notable exception within this clade is PxaTPS8 from *Pseudolarix amabilis* (golden larch), which utilizes GGPP to synthesize the diterpene pseudolarene [45]. In gymnosperms, they constitute the major components of oleoresins and are frequently emitted as volatiles. For instance, α-pinene, *β*-pinene, limonene, *β*-myrcene and *δ*-3-carene are among the most abundant monoterpenes in conifers [50]. To date, 54 monoTPSs have been characterized, collectively generating a vast array of products. Among these, approximately 20 pinene synthases have been identified, yielding three primary isomers: (+)-*α*-pinene, (−)-*α*-pinene, and (−)-*β*-pinene. Specifically, Pt30 [51], PbTPS-pin1, and PcTPS-pin1 [52] catalyze the conversion of GPP into (+)-*α*-pinene, whereas Ag3 [53], PbTPS-pin2 [52], and Pt1 [51] facilitate the production of (−)-*α*-pinene. Meanwhile, PbTPS-pin3 and PbTPS-pin4 [51] are specialized for (−)-*β*-pinene synthesis. Furthermore, 3-carene synthases, (−)-linalool synthases, and (−)-*β*-phellandrene synthases are widely distributed across gymnosperms lineages. For example, PbTPS-3car1 and PbTPS-phell1 [51] from *Pinus banksiana* catalyze the production of 3-carene and (−)-*β*-phellandrene from GPP, similar to PcTPS-3car1 and PcTPS-phell1 in *Pinus contorta*, as well as PsTPS-Car1 and PsTPS-Phel1 in *Picea sitchensis* [54]. A hallmark of these monoTPSs is their catalytic promiscuity, as most enzymes generate multiple products from a single substrate, thereby underpinning the immense structural diversity of gymnosperm monoterpenoids.

### 5.2. TPS-d2 Members Predominantly Encode Sesquiterpene Synthases

The TPS-d2 clade is primarily associated with sesquiterpene biosynthesis, with all 19 functionally characterized members identified to date utilizing farnesyl diphosphate (FPP) as a substrate. Notably, the total number of identified Class I sesTPSs remains lower than that of monoTPSs and diTPSs (Figure 5). This observation is consistent with the comparatively lower abundance of sesquiterpenes in gymnosperm oleoresins in comparison to mono- and diterpene constituents [55]. Among the characterized sesTPSs, bisabolene synthases represent a significant functional group. Specifically, PmeTPS3 from *Pseudotsuga menziesii* [56] catalyzes the conversion of FPP to *γ*-bisabolene. GbTPS2 (*Ginkgo bilaba*) [57], PaTPS-Bis (*Picea abies*) [58], PxaTPS5 (*Pseudolarix amabilis*) [45] and Ag1 (*Abies grandis*) facilitate the production of *α*-bisabolene from FPP. Furthermore, four *α*-farnesene synthases [51,54,59] predominantly synthesizing *α*-farnesene. And PmeTPS4 [56] from *Pseudotsuga menziesii* catalyzes the conversion of FPP to *β*-farnesene. A particularly intriguing case of catalytic versatility is PmTPS21 from *Pinus massoniana*, which functions as a bifunctional mono-/sesquiterpene synthase, yielding *α*-pinene from GPP and longifolene from FPP [60]. Additionally, other TPS-d2 members contribute to the structural breadth of gymnosperm resins by synthesizing intricate polycyclic scaffolds, such as longifolene, *α*-longipinene, *β*-cadinene, and humulene. Consistent with the monoTPSs, many sesTPSs are characterized by their multi-product specificity, orchestrating the formation of diverse terpenoid blends rather than a single compound. For instance, PgTPS-Hum predominantly produces *α*-humulene (~43%) and (E)-*β*-caryophyllene (~38%), alongside a suite of minor products [54]. These findings underscore the pivotal role of the TPS-d2 clade in expanding the chemical landscape of gymnosperms, particularly through enzymes that maintain stringent substrate specificity while achieving extraordinary structural diversity through product promiscuity.

### 5.3. TPS-d3 Clade Members Are Responsible for Encoding Diterpene Synthases

**Figure 6:**
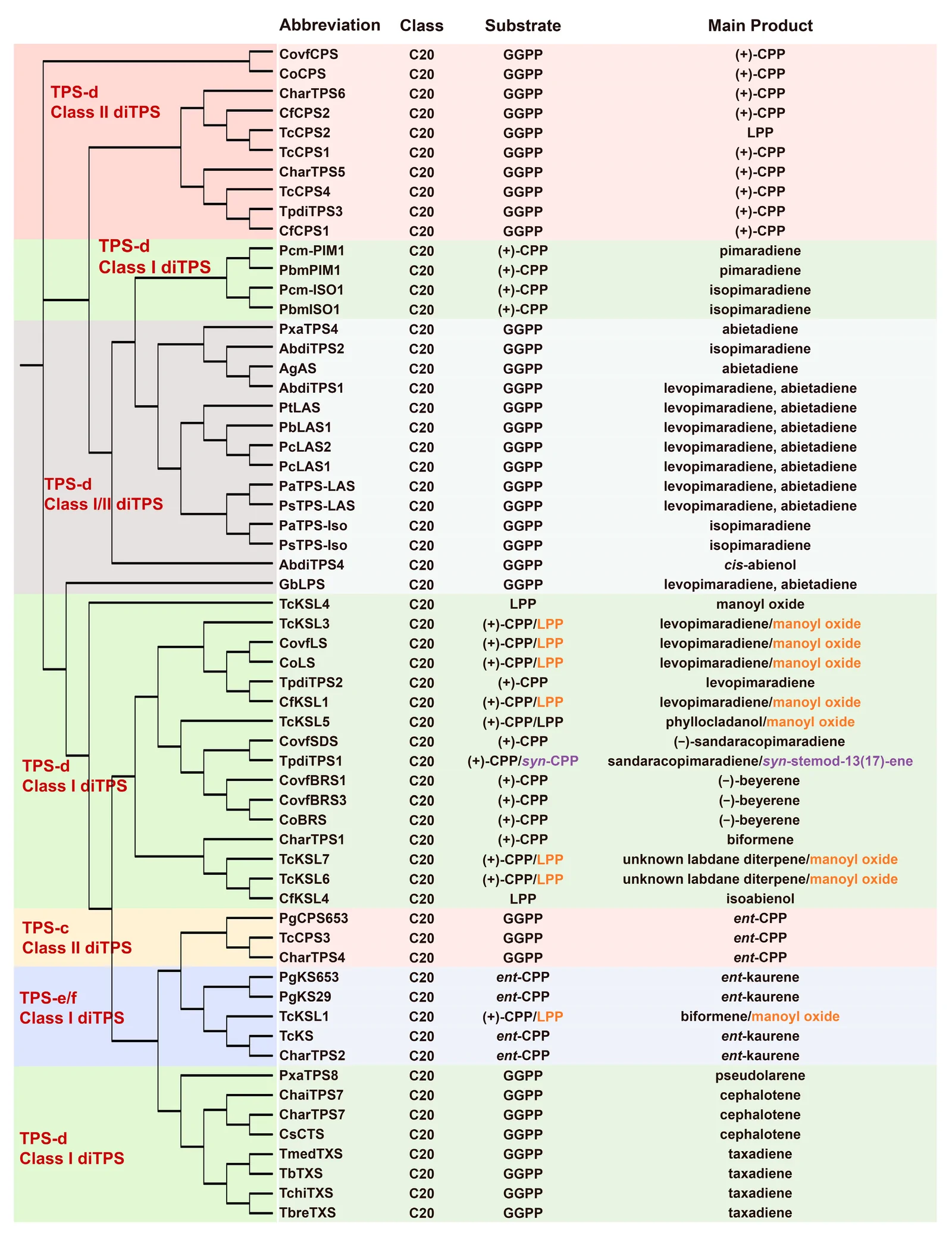
Phylogenetic analysis and functional characteristics of diTPSs in TPS-d genes. The left panel is a maximum-likelihood phylogenetic tree of diTPSs genes. In the right panel, Class I, Class II and Class I/II diTPS genes are distinguished by the different colors. The Class I/II diTPS, Class II diTPS, and Class I/II diTPS of the TPS-d genes are shaded in green, pink and gray, respectively. TPS-c and TPS e/f genes are shaded in yellow and purple, respectively. The four columns show the abbreviation, product class, substrate, and major product, respectively. Products catalyzed with the substrate LPP are shown in orange. Product generated from *syn*-CPP is shown in purple. GGPP, geranylgeranyl diphosphate; (+)-CPP, (+)-copalyl diphosphate; *ent*-CPP, *ent*-copalyl diphosphate; *syn*-CPP, *syn*-copalyl diphosphate; LPP, labda-13-en-8-ol diphosphate.

The TPS-d3 clade comprises exclusively diterpene synthases (diTPSs), which orchestrate the biosynthesis of specialized diterpenoids, such as the insect-resistant diterpene resin acids (DRAs). Based on their conserved motifs and catalytic mechanisms, these enzymes are categorized into three types (Class I, Class II, and Class I/II) (Figure 6).

Class II TPSs in the TPS-d3 clade, such as (+)-copalyl diphosphate synthases (CPSs) and labda-13-en-8-ol diphosphate synthases (LPSs), initiate the cyclization of GGPP into bicyclic intermediates. (+)-CPSs are predominantly found in the Cupressidae (e.g., CharTPS5/6 and CfCPS1/2) [39,61], whereas LPSs (producing LPP) appear more restricted, with TcCPS2 from *Taiwania cryptomerioides* being a documented example [40].

Subsequently, Class I diTPSs further transform these intermediates into specialized scaffolds. For instance, PIM-type TPSs (PcmPIM1) convert (+)-CPP to pimaradiene [62], while BRS-type TPSs catalyze the formation of beyerene [63]. Notably, some Class I enzymes exhibit substrate promiscuity; for example, TcKSL3 can transform (+)-CPP into levopimaradiene and LPP into manoyl oxide. Intriguingly, TpdiTPS1 from *Thuja plicata* is currently the only Class I enzyme known to utilize *syn*-CPP as a substrate in gymnosperms, despite the fact that no *syn*-CPS has yet been identified in this lineage [64]. However, certain Class I diTPSs have evolved to bypass bicyclic intermediates, directly converting GGPP into complex precursors. Notable examples include taxadiene synthases (TXSs), cephalotene synthases (CTS), and pseudolaratriene synthase (PxaTPS8), which provide the carbon skeletons for high-value compounds like paclitaxel, harringtonolide, and pseudolaric acid, respectively [39,45,65,66,67,68,69]. This specialized “one-step” mechanism stands in stark contrast to primary metabolism, where the biosynthesis of gibberellins remains tethered to the ancestral, modular organization of TPS-c (*ent*-CPS) and TPS-e/f (*ent*-KS) subfamilies across the plant kingdom.

Finally, bifunctional Class I/II diTPSs represent a specialized group that facilitates two-step cyclization cascades. Beyond the well-known abietadiene synthases (LAS), the recently identified AbdiTPS4 catalyzes the formation of *cis*-abienol [70]. This product shift is attributed to its Class II active site, which produces LPP instead of the typical (+)-CPP. From an evolutionary perspective, it is remarkable that while these bifunctional enzymes are prevalent in Pinaceae and Ginkgoaceae, they are conspicuously absent in Cupressaceae, suggesting a divergent evolutionary trajectory in domain architecture across gymnosperm families.

## 6. Ecological Roles of TPS-d Products in Defense and Chemical Communication

Terpenoids synthesized by the TPS-d subfamily constitute a sophisticated chemical defense system in gymnosperms, mediating responses to both biotic and abiotic stresses [15,55,71]. In addition to their defensive roles, volatile terpenoids also function as signaling compounds in ecological interactions. They play a key part in chemical communication between plants and insects, across different trophic levels, and between neighboring plants.

In response to biotic challenges, such as herbivory or pathogen attack, gymnosperms drastically upregulate the biosynthesis and accumulation of terpenoids, leading to the massive secretion of oleoresin. This viscous mixture serves a dual defensive role: it physically entraps or expels invading insects through its sticky consistency, while its chemical constituents exert potent toxicity. Upon exposure to air, volatile monoterpenes and sesquiterpenes evaporate, causing the remaining diterpene resin acids (DRAs) to polymerize and semi-crystallize. This process creates a resilient physical seal that protects the wounded site from further infection.

In Norway spruce (*Picea abies*), fungal inoculation consistently triggers the upregulation of terpene biosynthetic pathways, representing a significant metabolic reallocation toward defense [72]. Similarly, in Sitka spruce (*Picea sitchensis*), insect infestation induces the expression of several monoTPS genes, most notably those encoding (−)-pinene synthases [73,74]. Beyond spruces, studies in *Pinus brutia* have revealed elevated levels of tricyclene, camphene, and p-cymene in the phloem and needles of infested trees, reinforcing the role of induced terpenes as a central defensive tactic [75]. In white spruce (*Picea glauca*), specific monoterpenes contribute to foliage toxicity, effectively deterring defoliators and reducing herbivory pressure [76]. However, the ecological significance of volatile terpenoids extends beyond direct toxicity or deterrence, they also function as semiochemical cues in plant-insect interactions. For example, in mountain pine beetle-pine systems, host-derived monoterpenes are not only components of conifer defense but also can be exploited by bark beetles as host-location cues [71]. Likewise, in white spruce, the mixtures of α-pinene, *β*-pinene, limonene, and myrcene can stimulate feeding and oviposition by the spruce budworm. These findings suggest that compounds involved in plant defense may also provide information used by herbivores for host recognition and selection [77].

In addition, volatile terpenoids can mediate indirect defense by chemical communication. In Scots pine (*Pinus sylvestris*), oviposition by the pine sawfly (*Diprion pini*) induces changes in the blend of volatiles emitted by the host plant, and these emissions attract specialized egg parasitoids *Closterocerus ruforum* [78]. Chemical analyses have revealed that the resulting blend comprises mono- and sesquiterpenes, with an increased emission of the sesquiterpene (E)-*β*-farnesene following sawfly oviposition [79]. These findings demonstrate that conifer terpenoid emissions convey information to both herbivores and their natural enemies, thereby contributing to indirect plant defense. There is also emerging evidence that herbivore-induced volatiles play a role in plant-plant communication. In *Pinus sylvestris*, exposure to volatiles emitted by herbivore-attacked neighbors altered the subsequent volatile emissions, reducing later feeding damage by herbivores [80].

Based their ecological functions, volatile terpenoids also exhibit potential as insect deterrents and botanical pest-management agents. Monoterpene compounds such as *α*-pinene, limonene, camphor and citronellal have been reported to contribute to the repellent activity of plant essential oils against diverse insect pests. For example, essential oils dominated by mono- and sesquiterpenes showed contact, fumigant, and residual toxicity against the Indian meal moth *Plodia interpunctella*, a major pest of stored food products [81]. In addition, incorporation of terpene-rich essential oils into biodegradable packaging films has been shown to reduce larval penetration and infestation by *P. interpunctella*, illustrating the potential of volatile terpenoids in stored-product protection technologies [82]. These observations extend the functional significance of TPS-derived products beyond endogenous plant defense.

Beyond biotic interactions, TPS-d-mediated terpenoids are instrumental in mitigating abiotic stress. For instance, drought conditions have been shown to modulate terpene profiles and transcriptional regulation in slash pine (*Pinus elliottii*), where specific proteins in the terpene biosynthetic pathway are differentially expressed to cope with water deficit [83]. Furthermore, exposure to ultraviolet (UV) irradiation and cold stress significantly enhances terpene accumulation in species like *Ginkgo biloba*. This is achieved through the coordinated upregulation of key upstream genes (e.g., *GbDXS*, *GbGGPPS*, and *GbMVD*) alongside downstream specialized synthase like *GbLPS*, highlighting the multifaceted role of terpenoids as protective agents against environmental extremes [84].

## 7. Discussion and Future Prospects

Gymnosperms represent one of the four major lineages of land plants, and are divided into five subclasses including Cycadidae, Ginkgoidae, Cupressidae, Pinidae and Gnetidae [85]. Terpenoids represent the most abundant group of natural products and play essential roles in gymnosperm growth, development, and defense responses. Terpenoids are widely found and identified in gymnosperms. For instance, some monoterpenoids *β*-pinene, *β*-phellandrene, *α*-pinene, *δ*-3-carene and *α*-terpineol are found to constitute the essential oils in *Pinus* species [86]. *Ginkgo* terpenoids, primarily comprising ginkgolides and bilobalides, are distinctive to the *Ginkgo biloba* species [31,87]. Particularly, paclitaxel is a well-known anticancer terpenoid which is a diterpenoid in *Taxus* genus plants [88]. Additionally, the biosynthesis of these terpenoids in gymnosperms are extensively characterized [89,90,91,92,93,94,95,96,97,98,99,100,101,102,103]. Our review consolidated functional data for 134 TPS genes from 27 gymnosperm species, illustrating the significant expansion and diverse function of the TPS-d subfamily. However, the comprehensive summary of reported terpenoid genes in gymnosperms shows that the majority of validated genes were identified from the Pinales and Cupressales. Only three genes were characterized in the Ginkgoales, whilst no TPS genes have yet been functionally characterized in the Cycadales. Consequently, the evolutionary analysis here for the TPS-d subfamily based on the previous studies are predominantly driven by data from Pinales and Cupressales, suggesting there remains a significant gap in the identification of TPSs across Ginkgoales and Cycadales.

The species belonging to Cycadidae and Ginkgoidae represent ancient plant lineages, and certain terpenoids have been identified within these species [89]. Nevertheless, despite the discovery of terpenoids, there are 32 *TPS* genes that have been identified but not characterized in *Cycas panzhihuaensis*, while only three *TPS* genes have been characterized in *Ginkgo*. To further understand the evolution of TPS-d subfamily, more gymnosperms TPSs need to be identified and functional characterized. On the other hand, the modifications of certain gymnosperm-specific terpenoids remain unknown, such as ginkgolides and pseudolaric acids. Future work could focus on these unknown biosynthesis pathways, as complete biosynthesis pathway provide a deeper understanding of the species-specific enzymes and mechanisms in plants. The extensive publication of genome sequences has created an opportunity for studying TPS-d genes in a greater number.

The ecological functions of volatile monoterpenes and sesquiterpenes have been particularly well studied in conifers, especially members of the Pinaceae. These compounds not only participate in plant defense and chemical communications, also have practical importance in insect deterrence and crop or stored-product protection. Therefore, the application of terpenoids could ultimately facilitate the development of sustainable applications in insect deterrence, crop protection, and stored-product protection. In this context, continued identification and functional characterization of TPS-d enzymes will be important for understanding the biosynthetic basis of terpenoid diversity, providing a scalable production route of valuable mono- and sesquiterpenes.

## 8. Conclusions

In this review, we summarized 134 validated *TPS* genes from 27 gymnosperm species. Our summary of TPSs concerning evolutionary divergence and functionality across various families provides significant insights into the roles of terpenoids in facilitating plant defense. Our review reveals a significant expansion and functional divergence in the TPS-d subfamily, underlying the terpenoid diversity observed in gymnosperms. Furthermore, these gymnosperm-specific TPS-d members are responsible for the biosynthesis of specialized metabolites, which have been shown to play essential roles in plant resistance and chemical communication. This review provides a foundation for understanding the evolution and the diversity of the TPS-d subfamily, offering valuable insights for future research on terpene biosynthesis.

## Data Availability

The datasets generated and analyzed in the study are available in Supporting Information. The alignments and tree files are submitted to the TreeBase Datebase: http://purl.org/phylo/treebase/phylows/study/TB2:S32764 (accessed on 1 September 2026).

## Supplementary Materials

The following supporting information can be downloaded at https://www.mdpi.com/article/10.3390/biom16091318/s1, Table S1: The amount of TPSs characterized in gymnosperms; Table S2: Gymnosperm-specific monoTPSs used in phylogenetic analysis; Table S3: Gymnosperm-specific sesTPSs used in phylogenetic analysis; Table S4: Gymnosperm-specific diTPSs used in phylogenetic analysis; Table S5: TPSs of *Arabidopsis thaliana* and *Solanum lycopersicum* used in phylogenetic analysis; Figure S1: Phylogenetic analyses of gymnosperm TPSs and reported TPS from *Arabidopsis thaliana* and *Solanum lycopersicum*; Figure S2: The predicted protein structures of TPS-d3 diTPSs and TPS-d1 diTPSs PxaTPS8 in gymnosperms; Figure S3: The predicted protein structures of TPS-d2 sesTPSs in gymnosperms; Figure S4: The predicted protein structures of TPS-d1 monoTPSs in gymnosperms; Figure S5: Motif structures of monoTPS proteins in gymnosperms; Figure S6: Motif structures of sesTPS proteins in gymnosperms; Figure S7: Motif structures of diTPS proteins in gymnosperms.
