# Supplementary material for: Insight into TPS-d Subfamily: The Evolutionary and Functional Diversity in Gymnosperms
Source: Biomolecules. 2026 Sep 10;16(9):1318. doi: 10.3390/biom16091318 (PMC13604524; doi:10.3390/biom16091318)

Supplementary Materials

Insight into TPS-d Subfamily: The Evolutionary and Functional Diversity in Gymnosperms

The following Supporting Information is available for this article:

Table S1: The amount of TPSs characterized in gymnosperms.

Table S2: Gymnosperm-specific monoTPSs used in phylogenetic analysis.

Table S3: Gymnosperm-specific sesTPSs used in phylogenetic analysis.

Table S4: Gymnosperm-specific diTPSs used in phylogenetic analysis.

Table S5: TPSs of *Arabidopsis thaliana* and *Solanum lycopersicum* used in phylogenetic analysis.

Figure S1: Phylogenetic analyses of gymnosperm TPSs and reported TPS from *Arabidopsis thaliana* and *Solanum lycopersicum*.

Figure S2: The predicted protein structures of TPS-d3 diTPSs and TPS-d1 diTPSs PxaTPS8 in gymnosperms.

Figure S3: The predicted protein structures of TPS-d2 sesTPSs in gymnosperms.

Figure S4: The predicted protein structures of TPS-d1 monoTPSs in gymnosperms.

Figure S5: Motif structures of monoTPS proteins in gymnosperms.

Figure S6: Motif structures of sesTPS proteins in gymnosperms.

Figure S7: Motif structures of diTPS proteins in gymnosperms.

Table S1. The amount of TPSs characterized in gymnosperms.

order	Family	Species	The amount of TPSs	The amount of diTPSs	The amount of sesTPSs	The amount of monoTPSs	**References**
Pinales	Pinaceae	*Abies balsamea*	3	3	0	0	[70]
	Pinaceae	*Abies grandis*	11	1	3	7	[53, 90, 94, 95, 97]
	Pinaceae	*Pseudolarix amabilis*	3	2	1	0	[45, 98]
	Pinaceae	*Picea abies*	10	2	3	5	[58, 91]
	Pinaceae	*Pinus taeda*	5	1	1	3	[51, 99]
	Pinaceae	*Pinus banksiana*	12	3	0	9	[52, 62]
	Pinaceae	*Pinus contorta*	12	4	0	8	[52, 62]
	Pinaceae	*Picea glauca*	9	4	1	4	[41, 54]
	Pinaceae	*Picea sitchensis*	12	2	1	9	[54]
	Pinaceae	*Picea engelmannii* × *Picea glauca*	4	0	2	2	[54]
	Pinaceae	*Pseudotsuga menziesii*	4	0	2	2	[56]
	Pinaceae	*Pinus massoniana*	2	0	1	1	[60]
	Pinaceae	*Pinus elliottii*	3	0	0	3	[93]
Cupressales	Taxaceae	*Taxus media*	1	1	0	0	[67]
	Taxaceae	*Taxus baccata*	1	1	0	0	[68]
	Taxaceae	*Taxus brevifolia*	1	1	0	0	[65]
	Taxaceae	*Taxus chinensis*	1	1	0	0	[66]
	Taxaceae	*Taxus wallichiana*	1	0	1	0	[59]
	Taxodiaceae	*Taiwania cryptomerioides*	11	11	0	0	[40]

	Cephalotaxaceae	*Cephalotaxus sinensis*	1	1	0	0	[69]
	Cephalotaxaceae	*Cephalotaxus hainanensis*	1	1	0	0	[39]
	Cephalotaxaceae	*Cephalotaxus harringtonia*	6	6	0	0	[39]
	Cupressaceae	*Chamaecyparis formosensis*	6	4	1	1	[61, 92, 96]
	Cupressaceae	*Thuja plicata*	3	3	0	0	[64]
	Cupressaceae	*Chamaecyparis obtusa*	3	3	0	0	[63]
	Cupressaceae	*Chamaecyparis obtusa var. formosana*	5	5	0	0	[63]
Ginkgoales	Ginkgoaceae	*Ginkgo biloba*	3	1	2	0	[57, 100]

Table S2. Gymnosperm-specific monoTPSs used in phylogenetic analysis.

Abbreviation	mRNA Accession number	protein Accession number	Function	Species	Family	Reference
Ag8	AF139205.1	AAF61453.1	(−)-β-phellandrene synthase	*Abies grandis*	Pinaceae	[53]
Ag6	U87910	AAB70707.1	(−)-camphene synthase	*Abies grandis*	Pinaceae	[53]
Ag10	AF006193	AAB70907.1	(−)-limonene synthase	*Abies grandis*	Pinaceae	[90]
Ag11	AF139207	AAF61455.1	(−)-limonene/ (−)-α-pinene synthase	*Abies grandis*	Pinaceae	[53]
Ag2	U87908	AAB71084.1	myrcene synthase	*Abies grandis*	Pinaceae	[90]
Ag3	U87909	AAB71085.1	(−)-pinene synthase	*Abies grandis*	Pinaceae	[90]
Ag9	AF139206	AAF61454.1	terpinolene synthase	*Abies grandis*	Pinaceae	[53]
PaTPS-Car	AF461460	AAO73863.1	(+)-3-carene synthase	*Picea abies*	Pinaceae	[91]
PaTPS-Lim	AY473624	AAS47694.1	(−)-limonene synthase	*Picea abies*	Pinaceae	[58]
PaTPS-Myr	AY473626	AAS47696.1	myrcene synthase	*Picea abies*	Pinaceae	[58]
PaTPS-Lin	AY473623	AAS47693.1	(−)-linalool synthase	*Picea abies*	Pinaceae	[58]
PaTPS-Pin	AY473622	AAS47692.1	(−)-α/β-pinene synthase	*Picea abies*	Pinaceae	[58]
PgTPS-Pin2	HQ426154	ADZ45508.1	(−)-α/β-pinene synthase	*Picea glauca*	Pinaceae	[54]
PgTPS-Lin	HQ426151	ADZ45500.1	(−)-linalool synthase	*Picea glauca*	Pinaceae	[54]
PgTPS-Cin	HQ426160	ADZ45498.1	1,8-cineole synthase	*Picea glauca*	Pinaceae	[54]
PgeTPS-Car	HQ426152	ADZ45510.1	(+)-3-carene synthase	*Picea engelmannii x Picea glauca*	Pinaceae	[54]
PgeTPS-Cin	HQ426156	ADZ45497.1	1,8-cineole synthase	*Picea engelmannii x Picea glauca*	Pinaceae	[54]
PgTPS-Pin	HQ426153	ADZ45507.1	(−)-α/β-pinene synthase	*Picea glauca*	Pinaceae	[54]
PsTPS-Car	HQ426167	ADZ45511.1	(+)-3-carene synthase	*Picea sitchensis*	Pinaceae	[54]

PsTPS-Cin	HQ426165	ADZ45499.1	1,8-cineole synthase	*Picea sitchensis*	Pinaceae	[54]
PsTPS-Lin1	HQ426164	ADZ45501.1	(−)-linalool synthase	*Picea sitchensis*	Pinaceae	[54]
PsTPS-Lin2	HQ426168	ADZ45502.1	(−)-linalool synthase	*Picea sitchensis*	Pinaceae	[54]
PsTPS-Phel1	HQ426162	ADZ45504.1	(−)-β-phellandrene synthase	*Picea sitchensis*	Pinaceae	[54]
PsTPS-Phel2	HQ426169	ADZ45506.1	(−)-β-phellandrene synthase	*Picea sitchensis*	Pinaceae	[54]
PsTPS-Phel3	HQ426163	ADZ45505.1	(−)-β-phellandrene synthase	*Picea sitchensis*	Pinaceae	[54]
PsTPS-Phel4	HQ426159	ADZ45503.1	(−)-β-phellandrene synthase	*Picea sitchensis*	Pinaceae	[54]
PsTPS-Pin	HQ426166	ADZ45509.1	(−)-α/β-pinene synthase	*Picea sitchensis*	Pinaceae	[54]
PcTPS-pin1	JQ240295	AFU73847.1	(+)-α pinene synthase	*Pinus contorta*	Pinaceae	[52]
PcTPS-pin2	JQ240303	AFU73855.1	(+)-α pinene synthase	*Pinus contorta*	Pinaceae	[52]
PcTPS-phell1	JQ240301	AFU73853.1	(−)-β-phellandrene synthase	*Pinus contorta*	Pinaceae	[52]
PcTPS-phell2	JQ240300	AFU73852.1	(−)-β-phellandrene synthase	*Pinus contorta*	Pinaceae	[52]
PcTPS-camp/pin	JQ240299	AFU73851.1	(−)-camphene / (+)-α-pinene synthase	*Pinus contorta*	Pinaceae	[52]
PcTPS-pin3	JQ240293	AFU73845.1	(−)-β-pinene synthase	*Pinus contorta*	Pinaceae	[52]
PcTPS-terp	JQ240309	AFU73861.1	α-terpineol /1,8-cineole synthase	*Pinus contorta*	Pinaceae	[52]
PcTPS-3car1	JQ240307	AFU73859.1	(+)-3-carene synthase	*Pinus contorta*	Pinaceae	[52]
PbTPS-pin1	JQ240298	AFU73850.1	(+)-α pinene synthase	*Pinus banksiana*	Pinaceae	[52]
PbTPS-pin2	JQ240304	AFU73856.1	(−)-α pinene synthase	*Pinus banksiana*	Pinaceae	[52]
PbTPS-phell1	JQ240302	AFU73854.1	(−)-β-phellandrene synthase	*Pinus banksiana*	Pinaceae	[52]
PbTPS-pin3	JQ240291	AFU73843.1	(−)-β-pinene synthase	*Pinus banksiana*	Pinaceae	[52]
PbTPS-pin4	JQ240292	AFU73844.1	(−)-β-pinene synthase	*Pinus banksiana*	Pinaceae	[52]
PbTPS-pin5	JQ240290	AFU73842.1	(−)-α/β-pinene synthase	*Pinus banksiana*	Pinaceae	[52]
PbTPS-terp	JQ240308	AFU73860.1	α-terpineol synthase	*Pinus banksiana*	Pinaceae	[52]
PbTPS-3car1	JQ240305	AFU73857.1	(+)-3-carene synthase	*Pinus banksiana*	Pinaceae	[52]

PbTPS-3car2	JQ240306	AFU73858.1	(+)-3-carene synthase	*Pinus banksiana*	Pinaceae	[52]
Pt1	AF543527	AAO61225.1	(−)-*α* pinene synthase	*Pinus taeda*	Pinaceae	[51]
Pt30	AF543530	AAO61228.1	(+)-*α* pinene synthase	*Pinus taeda*	Pinaceae	[51]
Pt10	AF543529	AAO61227.1	*α*-terpineol synthase	*Pinus taeda*	Pinaceae	[51]
PmeTPS1	AY906869	AAX07267.1	(−)-*α*-pinene/ (−)-camphene synthase	*Pseudotsuga menziesii (Abies menziesii)*	Pinaceae	[56]
PmeTPS2	AY906866	AAX07264.1	terpinolene synthase	*Pseudotsuga menziesii (Abies menziesii)*	Pinaceae	[56]
Cf-pin	EU099434	ABW80964.1	*α*-pinene synthase	*Chamaecyparis formosensis*	Cupressaceae	[92]
PmTPS21	MN832901	QLH02025	*α*-pinene synthase	*Pinus massoniana*	Pinaceae	[60]
Pec308173	not published	not published	*β*-pinene synthase	*Pinus elliottii*	Pinaceae	[93]
Pec348396	not published	not published	*α*-pinene synthase	*Pinus elliottii*	Pinaceae	[93]
Pec308177	not published	not published	*α*-pinene synthase	*Pinus elliottii*	Pinaceae	[93]

Table S3. Gymnosperm-specific sesTPSs used in phylogenetic analysis.

Abbreviation	mRNA Accession number	protein Accession number	Function	Species	Family	Reference
PmTPS4	MN832900	QLH02024	longifolene synthase	*Pinus massoniana*	Pinaceae	[60]
Ag1	AF006195	AAC24192.1	*E*-α-bisabolene synthase	*Abies grandis*	Pinaceae	[94]
Ag4	U92266.1	AAC05727.1	δ-selinene synthase	*Abies grandis*	Pinaceae	[95]
Ag5	U92267	AAC05728.1	γ-humulene synthase	*Abies grandis*	Pinaceae	[95]
PaTPS-Lon	AY473625	AAS47695.1	longifolene synthase	*Picea abies*	Pinaceae	[58]
PaTPS-Far	AY473627	AAS47697.1	*E*,*E*-α-farnesene synthase	*Picea abies*	Pinaceae	[58]
PaTPS-Bis	AY473619	AAS47689.1	*E*-α-bisabolene synthase	*Picea abies*	Pinaceae	[58]
PgeTPS-Far/Oci	HQ426157	ADZ45514.1	*E*,*E*-α-farnesene/*E*-β-ocimene synthase	*Picea engelmannii x Picea glauca*	Pinaceae	[54]
PgTPS-Hum	HQ426155	ADZ45513.1	α-humulene synthase	*Picea glauca*	Pinaceae	[54]
PgeTPS-Lon	HQ426158	ADZ45515.1	longifolene synthase	*Picea engelmannii x Picea glauca*	Pinaceae	[54]
PsTPS-Lonp	HQ426161	ADZ45516.1	α-longipinene synthase	*Picea sitchensis*	Pinaceae	[54]
Pt5	AF543528	AAO61226.1	*E*,*E*-α-farnesene synthase	*Pinus taeda*	Pinaceae	[51]
PxaTPS5	KU685113	APT40485.1	*E*-α-bisabolene synthase	*Pseudolarix amabilis*	Pinaceae	[45]
PmeTPS3	AY906868	AAX07266.1	*E*-γ-bisabolene synthase	*Pseudotsuga menziesii (Abies menziesii)*	Pinaceae	[56]
PmeTPS4	AY906867	AAX07265.1	*E*-β-farnesene synthase	*Pseudotsuga menziesii (Abies menziesii)*	Pinaceae	[56]
Cf-Cad	JN715077	AFJ23663.1	β-cadinene synthase	*Chamaecyparis formosensis*	Cupressaceae	[96]

GbTPS1	KM248383	AIU94289.1	*E,E*-α-farnesene synthase	*Ginkgo biloba*	Ginkgoacea e	[57]
GbTPS2	KM248384	AIU94290.1	*E*-α-bisabolene synthase	*Ginkgo biloba*	Ginkgoacea e	[57]
TwSTPS1	PP496059	WXG22020.1	(3*Z*,6*E*)-α-farnesene synthase	*Taxus wallichiana*	Taxaceae	[59]

Table S4. Gymnosperm-specific diTPSs used in phylogenetic analysis.

Abbreviation	mRNA Accession number	protein Accession number	Function	Type	Species	Family	Reference
CsCTS	OQ305433	WMI31575.1	chloroplast cephalotene synthase	Class Ⅰ	*Cephalotaxus sinensis*	Cephalotaxaceae	[69]
ChaiTPS7	OQ378948	WJJ50354	chloroplast cephalotene synthase	Class Ⅰ	*Cephalotaxus hainanensis*	Cephalotaxaceae	[39]
CharTPS1	OQ378949	WJJ50355	biformene synthase	Class Ⅰ	*Cephalotaxus harringtonia*	Cephalotaxaceae	[39]
CharTPS2	OQ378950	WJJ50356	*ent*-kaurene synthase	Class Ⅰ	*Cephalotaxus harringtonia*	Cephalotaxaceae	[39]
CharTPS4	OQ378952	WJJ50358	*ent*-CPP synthase	Class Ⅱ	*Cephalotaxus harringtonia*	Cephalotaxaceae	[39]
CharTPS5	OQ378953	WJJ50359	(+)-CPP synthases	Class Ⅱ	*Cephalotaxus harringtonia*	Cephalotaxaceae	[39]
CharTPS6	OQ378954	WJJ50360	(+)-CPP synthases	Class Ⅱ	*Cephalotaxus harringtonia*	Cephalotaxaceae	[39]
CharTPS7	OQ378955	WJJ50361	chloroplast cephalotene synthase	Class Ⅰ	*Cephalotaxus harringtonia*	Cephalotaxaceae	[39]
AbdiTPS1	JN254805	AEL99950	levopimaradiene/abietadiene synthase	Class Ⅰ/Ⅱ	*Abies balsamea*	Pinaceae	[70]
AbdiTPS2	JN254806	AEL99951	isopimaradiene synthases	Class Ⅰ/Ⅱ	*Abies balsamea*	Pinaceae	[70]
AbdiTPS4	JN254808	AEL99953	cis-abienol synthases	Class Ⅰ/Ⅱ	*Abies balsamea*	Pinaceae	[70]
AgAS	U50768	AAB05407	(+)-CPP/ abietadiene synthase	Class Ⅰ/Ⅱ	*Abies grandis*	Pinaceae	[97]
PxaTPS4	KC702398	AGN70885	levopimaradiene/abietadiene synthase	Class Ⅰ/Ⅱ	*Pseudolarix amabilis*	Pinaceae	[98]
PxaTPS8	KU685114	APT40486	pseudolarene synthase	Class Ⅰ	*Pseudolarix amabilis*	Pinaceae	[45]

PaTPS-Iso	AY473620	AAS47690.2	isopimaradiene synthase	Class Ⅰ/Ⅱ	*Picea abies*	Pinaceae	[58]
PaTPS-LAS	AY473621	AAS47691.1	levopimaradiene/abietadiene synthase	Class Ⅰ/Ⅱ	*Picea abies*	Pinaceae	[58]
PtLAS	AY779541	AAX07435.1	levopimaradiene/abietadiene synthase	Class Ⅰ/Ⅱ	*Pinus taeda*	Pinaceae	[99]
Pb-LAS1	JQ240312	AFU73864.1	levopimaradiene/abietadiene synthase	Class Ⅰ/Ⅱ	*Pinus banksiana*	Pinaceae	[62]
Pbm-PIM1	JQ240315	AFU73867.1	pimaradiene synthase	Class Ⅰ	*Pinus banksiana*	Pinaceae	[62]
Pbm-ISO1	JQ240313	AFU73865.1	isopimaradiene synthase	Class Ⅰ	*Pinus banksiana*	Pinaceae	[62]
Pc-LAS1	JQ240310	AFU73862.1	levopimaradiene/abietadiene synthase	Class Ⅰ/Ⅱ	*Pinus contorta*	Pinaceae	[62]
Pc-LAS2	JQ240311	AFU73863.1	levopimaradiene/abietadiene synthase	Class Ⅰ/Ⅱ	*Pinus contorta*	Pinaceae	[62]
Pcm-PIM1	JQ240316	AFU73868.1	pimaradiene synthase	Class Ⅰ	*Pinus contorta*	Pinaceae	[62]
Pcm-ISO1	JQ240314	AFU73866.1	isopimaradiene synthase	Class Ⅰ	*Pinus contorta*	Pinaceae	[62]
CfCPS1	MT275974	QWV53997	(+)-CPP synthases	Class Ⅱ	*Chamaecyparis formosensis*	Cupressaceae	[61]
CfCPS2	MT275975	QWV53998.1	(+)-CPP synthases	Class Ⅱ	*Chamaecyparis formosensis*	Cupressaceae	[61]
CfKSL1	MT275969	QWV53992.1	levopimaradiene synthase	Class Ⅰ	*Chamaecyparis formosensis*	Cupressaceae	[61]
CfKSL4	MT275972	QWV53995.1	isoabienol synthase	Class Ⅰ	*Chamaecyparis formosensis*	Cupressaceae	[61]
TpdiTPS1	MT468207	QND75951.1	sandaracopimaradiene/syn-stemod-13(17)-ene synthase synthase	Class Ⅰ	*Thuja plicata*	Cupressaceae	[64]
TpdiTPS2	MT468208	QND75952.1	levopimaradiene synthases	Class Ⅰ	*Thuja plicata*	Cupressaceae	[64]
TpdiTPS3	MT468209	QND75953.1	(+)-CPP synthases	Class Ⅱ	*Thuja plicata*	Cupressaceae	[64]
TcCPS4	JN587309	AFE61356.1	(+)-CPP synthase	Class Ⅱ	*Taiwania cryptomerioides*	Cupressaceae	[40]

TcCPS1	KT588482	AOG18228.1	(+)-CPP synthase	Class II	*Taiwania cryptomerioides*	Cupressaceae	[40]
TcCPS2	KT588483	AOG18229.1	LPP synthase	Class II	*Taiwania cryptomerioides*	Cupressaceae	[40]
TcCPS3	KT588491	AOG18237.1	*ent*-CPP synthase	Class II	*Taiwania cryptomerioides*	Cupressaceae	[40]
TcKSL4	KT588486	AOG18232.1	manoyl oxide synthase	Class I	*Taiwania cryptomerioides*	Cupressaceae	[40]
TcKSL3	GU575291	ADL14246.1	levopimaradiene/abietadiene synthase	Class I	*Taiwania cryptomerioides*	Cupressaceae	[40]
TcKSL5	KT588490	AOG18236.1	phyllocladanol synthase	Class I	*Taiwania cryptomerioides*	Cupressaceae	[40]
TcKSL6	KT588487	AOG18233.1	manoyl oxide synthase	Class I	*Taiwania cryptomerioides*	Cupressaceae	[40]
TcKSL7	KT588488	AOG18234.1	manoyl oxide synthase	Class I	*Taiwania cryptomerioides*	Cupressaceae	[40]
TcKSL1	KT588484	AOG18230.1	biformene synthase	Class I	*Taiwania cryptomerioides*	Cupressaceae	[40]
TcKS	KT588489	AOG18235.1	*ent*-kaurene synthase	Class I	*Taiwania cryptomerioides*	Cupressaceae	[40]
GbLPS	AF331704	AAL09965.1	levopimaradiene/abietadiene synthase	Class I / II	*Ginkgo biloba*	Ginkgoaceae	[100]
CoCPS	ON060907	WVX33989.1	(+)-CPP synthase	Class II	*Chamaecyparis obtusa*	Cupressaceae	[63]
CovfCPS	ON060905	WVX33987.1	(+)-CPP synthase	Class II	*Chamaecyparis obtusa* var. *formosana*	Cupressaceae	[63]
CoLS	ON060908	WVX33990.1	levopimaradiene synthase	Class I	*Chamaecyparis obtusa*	Cupressaceae	[63]
CovfLS	ON060906	WVX33988.1	levopimaradiene synthase	Class I	*Chamaecyparis obtusa* var. *formosana*	Cupressaceae	[63]
CoBRS	OR359412	WZC45483.1	(−)-beyerene synthase	Class I	*Chamaecyparis obtusa*	Cupressaceae	[63]
CovfBRS1	OR359413	WZC45484.1	(−)-beyerene synthase	Class I	*Chamaecyparis obtusa* var. *formosana*	Cupressaceae	[63]
CovfBRS3	OR359415	WZC45486.1	(−)-beyerene synthase	Class I	*Chamaecyparis obtusa* var. *formosana*	Cupressaceae	[63]

CovfSDS	OR359416	WZC45487.1	(−)-sandaracopimaradiene synthase	Class Ⅰ	*Chamaecyparis obtusa* var. *formosana*	Cupressaceae	[63]
PgKS29	GU144565	ADB55711.1	*ent*-kaurene synthase	Class Ⅰ	*Picea glauca*	Pinaceae	[41]
PgKS653	GU045756	ADB55708.1	*ent*-kaurene synthase	Class Ⅰ	*Picea glauca*	Pinaceae	[41]
PgCPS29	GU059904.1	ACY25274.1	*ent*-CPP synthase	Class Ⅱ	*Picea glauca*	Pinaceae	[41]
PgCPS653	GU045755	ADB55707.1	*ent*-CPP synthase	Class Ⅱ	*Picea glauca*	Pinaceae	[41]
PsTPS-ISO	HQ426150	ADZ45512.1	isopimaradiene synthase	Class Ⅰ/Ⅱ	*Picea sitchensis*	Pinaceae	[54]
PsTPS-LAS	HQ426170	ADZ45517.1	levopimaradiene/abietadiene synthase	Class Ⅰ/Ⅱ	*Picea sitchensis*	Pinaceae	[54]
TmedTXS	AY461450.2	AAS18603.1	taxadiene synthase	Class Ⅰ	*Taxus media*	Pinaceae	[67]
TbTXS	AY424738	AAR02861.1	taxadiene synthase	Class Ⅰ	*Taxus baccata*	Pinaceae	[68]
TbreTXS	U48796	AAC49310.1	taxadiene synthase	Class Ⅰ	*Taxus brevifolia*	Pinaceae	[65]
TchiTXS	AY007207.1	AAG02257.1	taxadiene synthase	Class Ⅰ	*Taxus chinensis*	Pinaceae	[66]

Table S5. TPSs of *Arabidopsis thaliana* and *Solanum lycopersicum* used in phylogenetic analysis.

Abbreviation	mRNA Accession number	protein Accession number	TPS subfamily	References
AtTPS-GA1	U11034.1	AAA53632.1	TPS-c	[101]
AtTPS10	AF178535.1	AAG09310.1	TPS-b	[102]
AtTPS03	AY151086.1	AAN65379.1	TPS-b	[103]
SolyTPS3	JN408284.1	AEM05853.1	TPS-b	[43]
SolyTPS4	JN408285.1	AEM05854.1	TPS-b	[43]
SolyTPS5	JN408286.1	AEM05855.1	TPS-b	[43]
SolyTPS7	JN408287.1	AEM05856.1	TPS-b	[43]
SolyTPS10	JN412093.1	AEP82784.1	TPS-a	[43]
SolyTPS20	JN412071.1	AEP82763.1	TPS-e/f	[43]
SolyTPS21	JN412087.1	AEP82779.1	TPS-e/f	[43]
SolyTPS24	JN412086.1	AEP82778.1	TPS-e/f	[43]
SolyTPS28	JN412083.1	AEP82775.1	TPS-a	[43]
SolyTPS33	JN412080.1	AEP82772.1	TPS-a	[43]
SolyTPS37	JN412077.1	AEP82769.1	TPS-g	[43]
SolyTPS39	JN412075.1	AEP82767.1	TPS-g	[43]
SolyTPS40	JN412074.1	AEP82766.1	TPS-c	[43]
SolyTPS41	JN412073.1	AEP82765.1	TPS-c	[43]
SolyTPS51	MN366354.1	QIU80844.1	TPS-a	[43]
SolyTPS52	MN366355.1	QIU80845.1	TPS-a	[43]

Figure S1. Phylogenetic analyses of gymnosperm TPSs and reported TPS from *Arabidopsis thaliana* and *Solanum lycopersicum*.

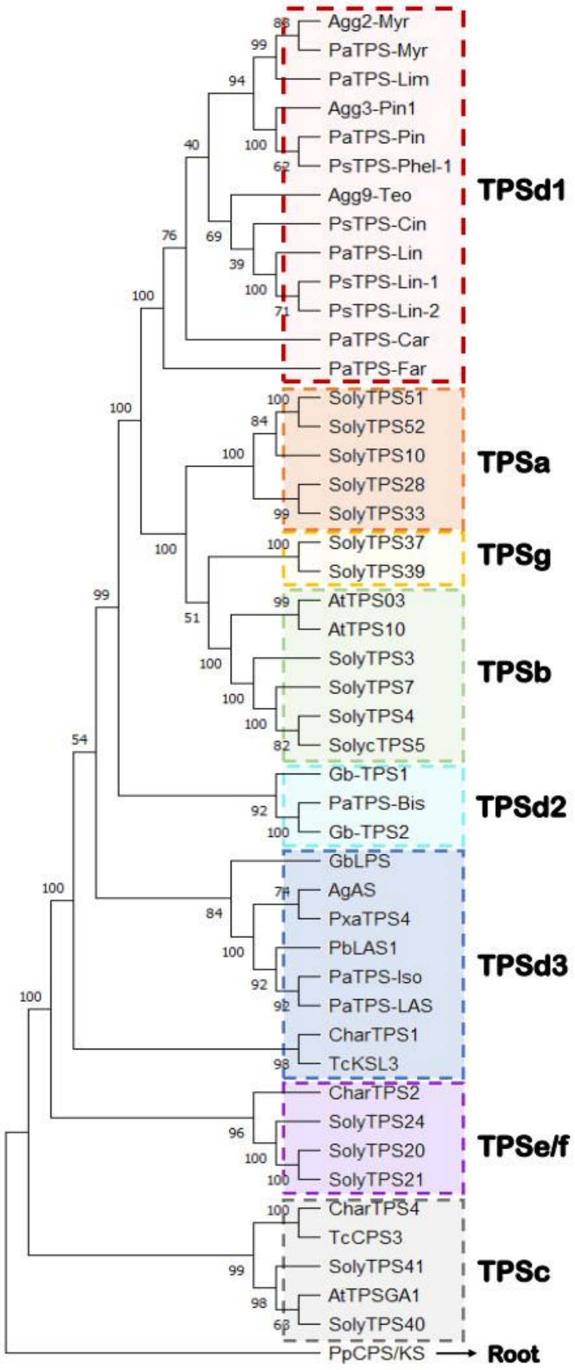

Figure S2. The predicted protein structures of TPS-d3 diTPSs and TPS-d1 diTPSs PxaTPS8 in gymnosperms. AgAs is a diTPS with a known crystal structure and a $\gamma\beta\alpha$ tridomain architecture; the other 59 diTPSs also retain this $\gamma\beta\alpha$ tridomain configuration. The sequences of these diTPSs were listed in Table S2.

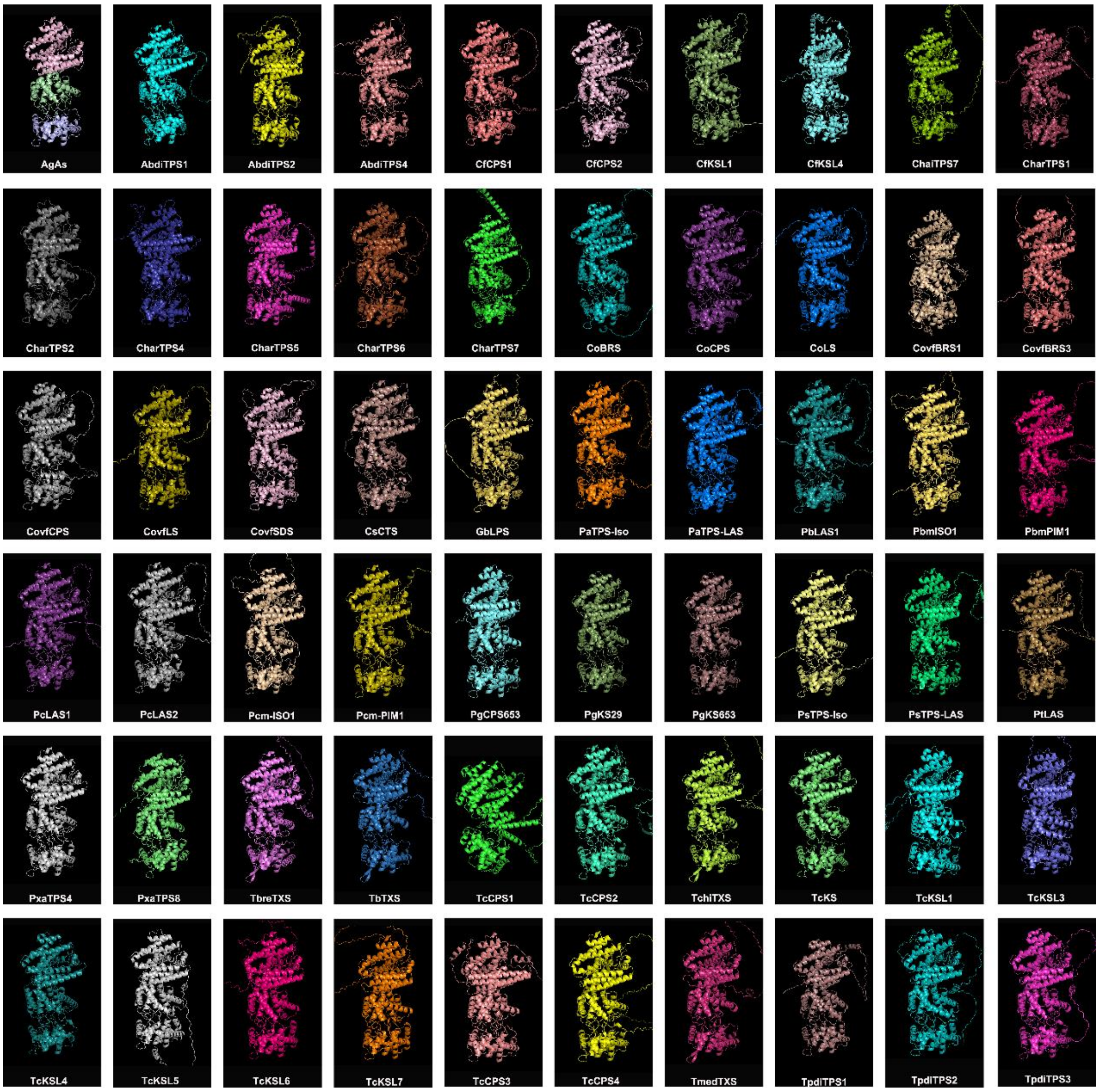

Figure S3. The predicted protein structures of TPS-d2 sesTPSs in gymnosperms. Most of TPS-d2 sesTPSs have lost the γ domain, resulting in a $\beta\alpha$ bidomain structure. Seven sesTPSs still retain the $\gamma\beta\alpha$ tridomain structure, namely Ag1, GbTPS1, GbTPS2, PaTPS-Bis, PmeTPS3, PmeTPS4 and PxaTPS5. The sequences of these sesTPSs were listed in Table S3.

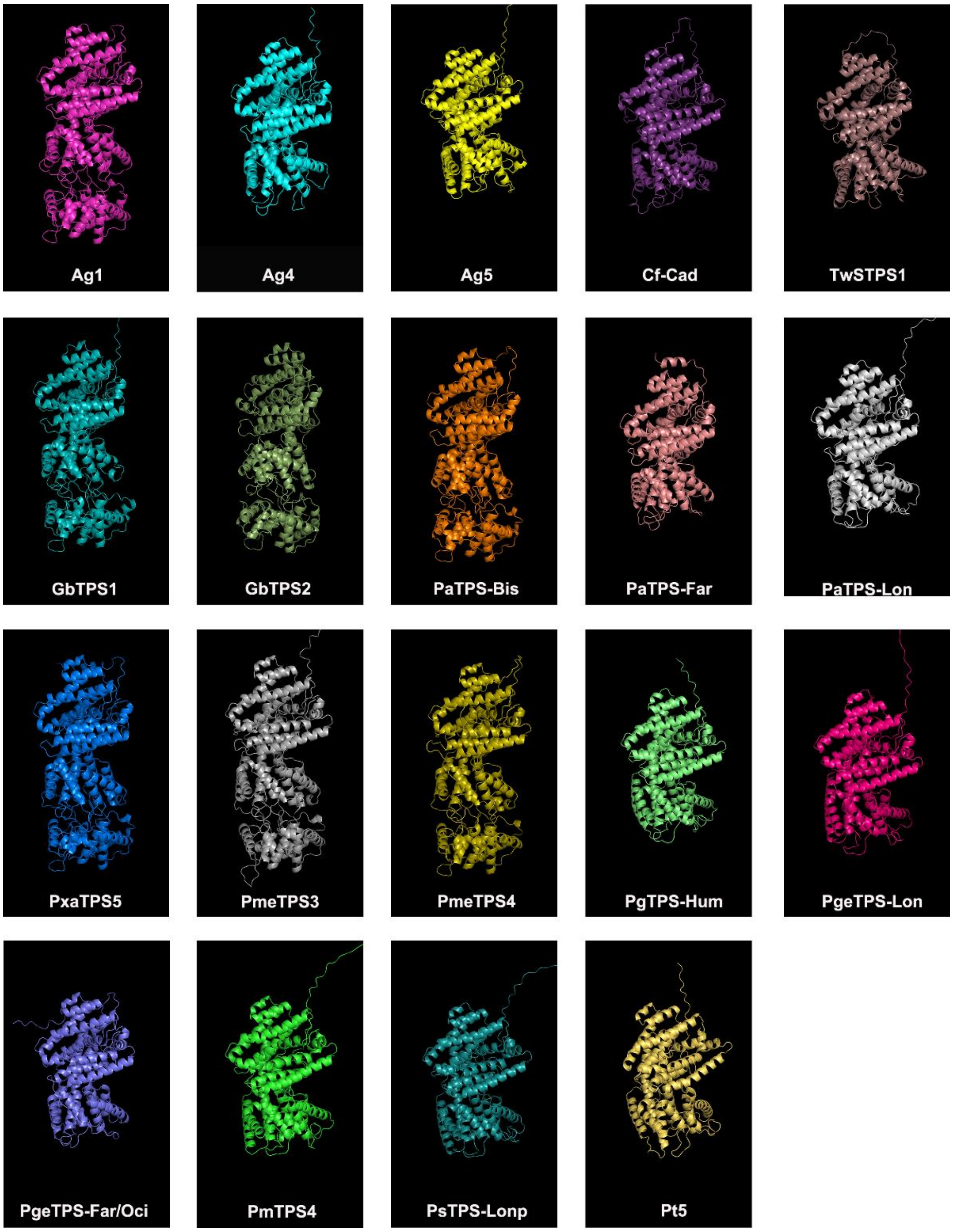

Figure S4. The predicted protein structures of TPS-d1 monoTPSs in gymnosperms. All TPS-d1 monoTPSs have lost the γ domain, resulting in a $\beta\alpha$ bidomain structure. The sequences of these monoTPSs were listed in Table S4.

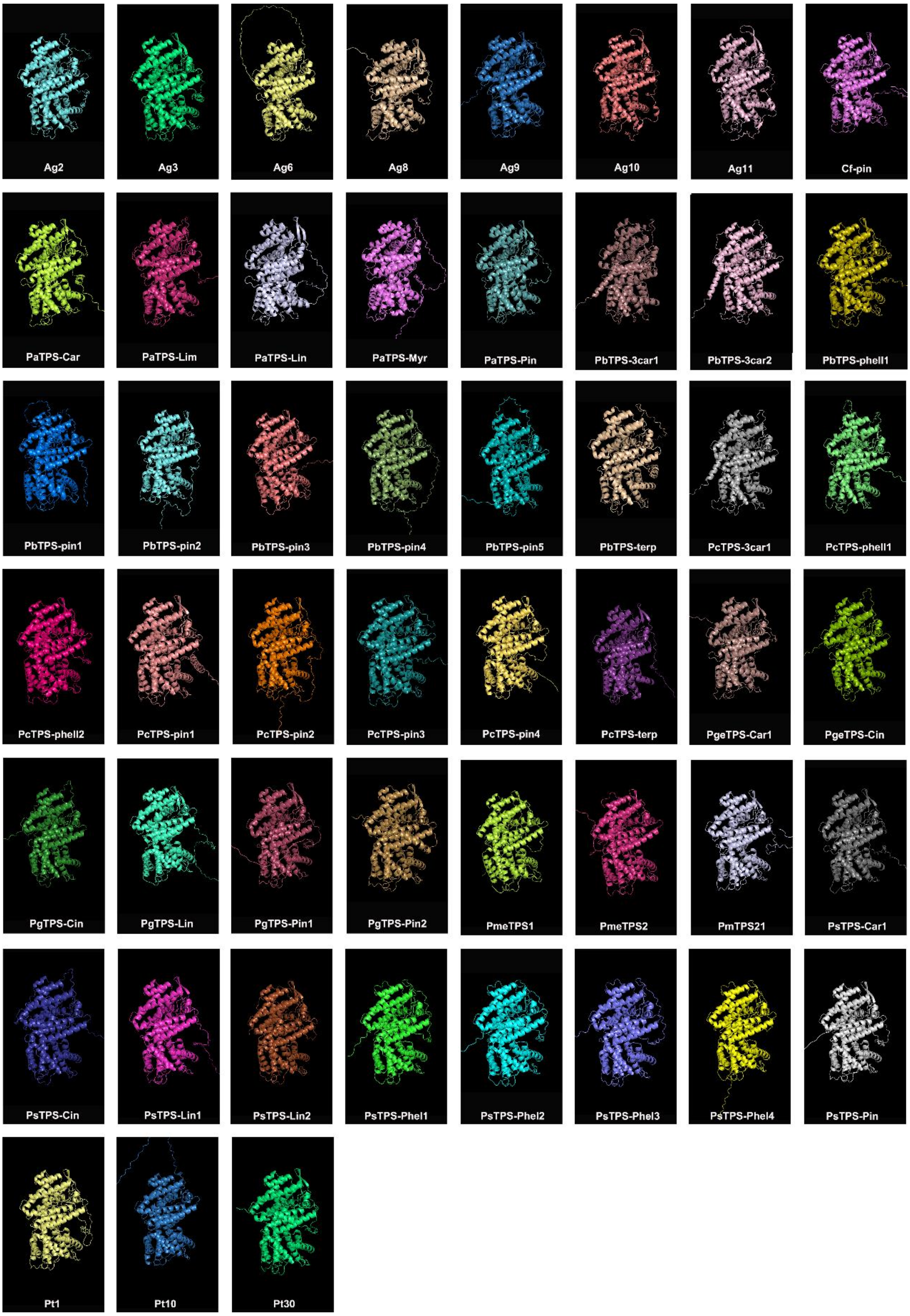

Figure S5. Motif structures of monoTPS proteins in gymnosperms. Different color blocks represent distinct motifs; the arrow indicates RR(X)8W and DDXXD motifs in monoterpene synthases.

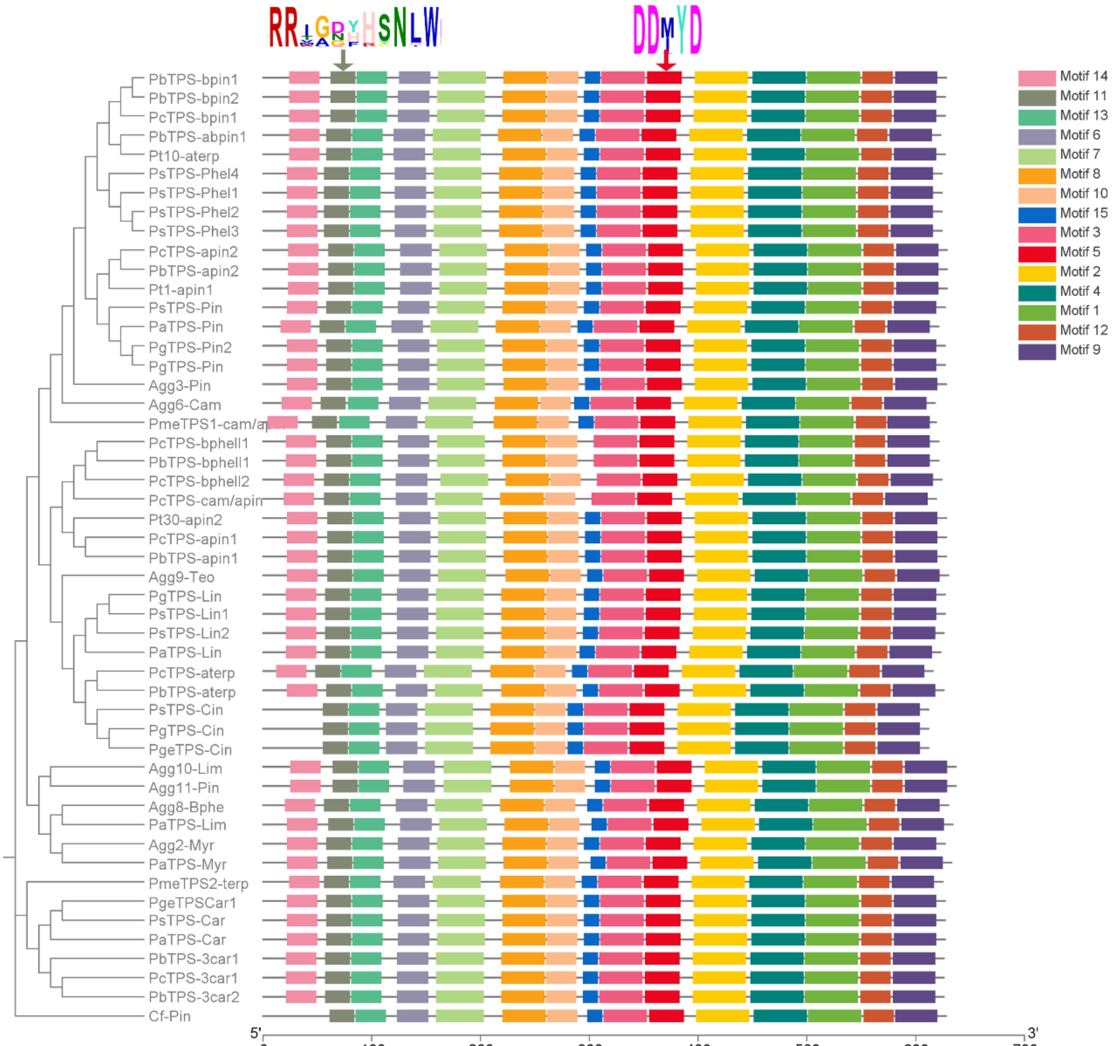

Figure S6. Motif structures of sesTPS proteins in gymnosperms. Different color blocks represent distinct motif; the arrow indicates RR(X)8W and DDXXD motifs in sesquiterpene synthases.

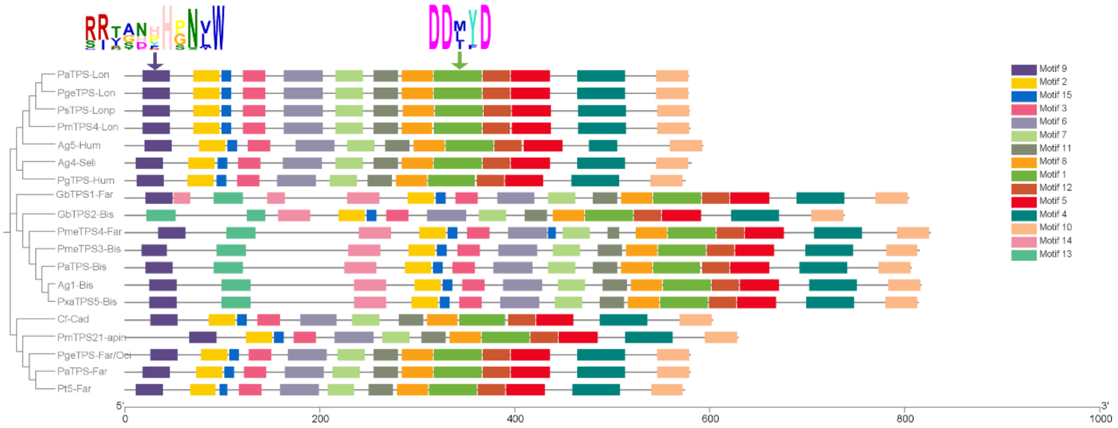

Figure S7. Motif structures of diTPS proteins in gymnosperms. Different color blocks represent distinct motif; the arrow indicates DXDD and DDXXD motifs in diterpene synthases.

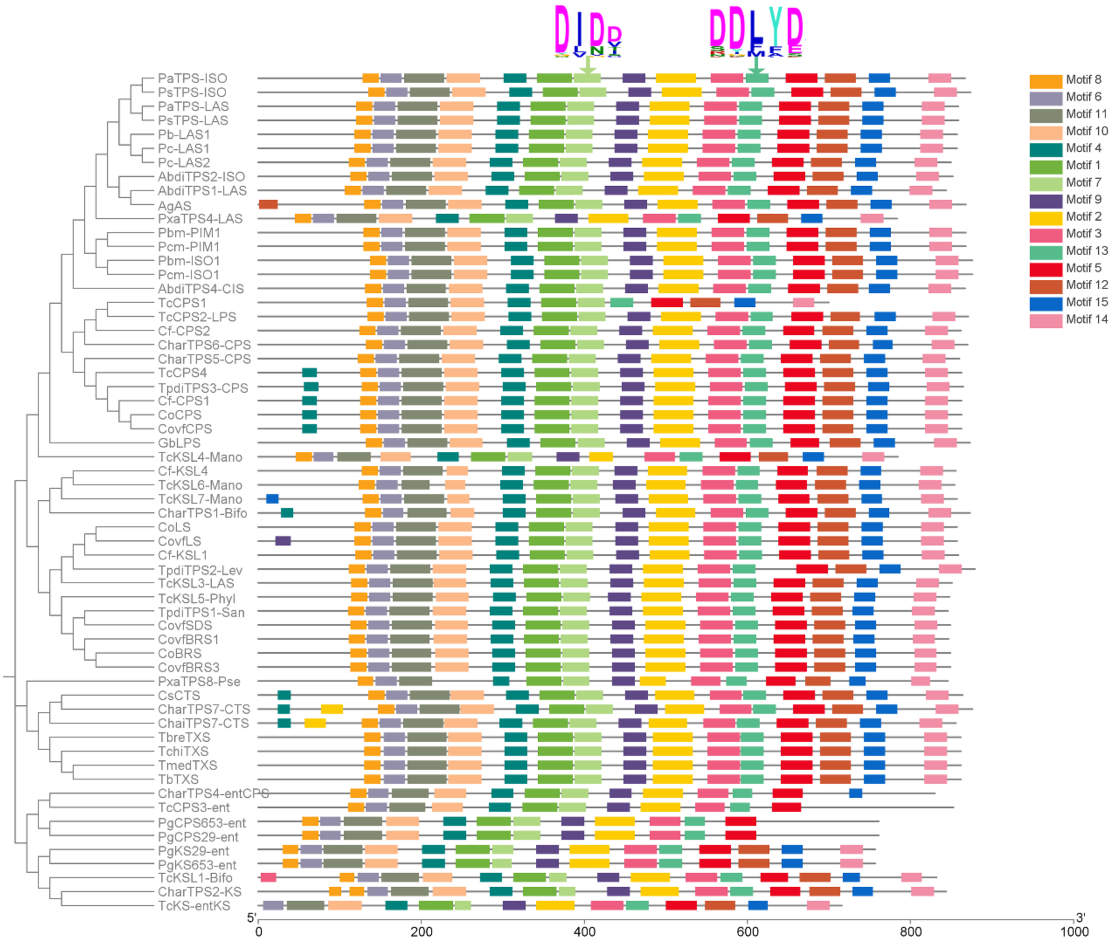

Supplement: Supplementary file 1 [file biomolecules-16-01318-s001.zip › biomolecules-4527099-supplementaryv7.pdf]
